# Alliance Between Conifer Trees and Endophytic Fungi Against Insect Defoliators

**DOI:** 10.1111/pce.15503

**Published:** 2025-04-01

**Authors:** Aziz Ullah, Ateeq Shah, Shih‐hsuan (Ethan) Chen, Aftab Shah, Jean C. Rodriguez‐Ramos, Rashaduz Zaman, Nadir Erbilgin

**Affiliations:** ^1^ Department of Renewable Resources University of Alberta Edmonton Alberta Canada

**Keywords:** *Choristoneura fumiferana*, conifer defences, fungal volatile organic compounds, monoterpenes and sesquiterpenes, *Picea glauca*

## Abstract

Fungal endophytes can alter plant resistance against herbivores by indirectly influencing plant secondary metabolism or through direct effects of their own metabolism. However, the role of fungal endophytes in conifer defences to insect herbivores remains largely unknown. We characterised the endophytic fungal communities and terpene concentrations of 30 white spruce families across two sites. We determined the effects of fungal endophytes on a defoliating insect, eastern spruce budworm, by testing the budworm responses to media amended with fungal endophytes or exposing them to their volatile organic compounds. We further examined whether the changes in the endophytic fungal communities and abundance alter the terpene concentrations of white spruce by inoculating seedlings with endophytic fungi. Terpene and fungal community compositions in mature trees varied among families and sites. The bioassays showed fungal endophytes can kill budworms or reduce their fitness due to the toxicity of fungal mycelium or volatile compounds. The inoculation experiments demonstrated that the changes in fungal communities and abundance can alter the terpene concentrations in seedlings. We developed a “Plant Partnership Hypothesis” to reflect the role of fungal endophytes in plant resistance to insect herbivores, demonstrating a co‐evolutionary relationship among fungal endophytes, tree defences, and insect herbivores.

## Summary statement

We found that the fungal endophytes in white spruce (*Picea glauca*) provide anti‐herbivory protection to eastern spruce budworm and that higher fungal abundance correspond to the increased terpene production in foliage, highlighting the co‐evolutionary importance of these fungi in conifer defences.

## 1 Introduction

Endophytes are microorganisms, primarily consisting of fungi and bacteria, inhabiting plant tissues without causing any discernible harm (Wilson 1995; Hardoim et al. 2015). Plant endophytes are transferred horizontally, through sexual or asexual spores, or vertically, from parent to offspring via host seeds (Clay and Schardl 2002). They can improve the growth and survival of host plants by increasing nutrient acquisition, contributing to tolerance to abiotic stressors, and enhancing herbivore resistance (Wilson 1995; Rodriguez et al. 2009; Hardoim et al. 2015). Fungal endophytes, in particular, influence plant defence through multiple mechanisms, including the production of toxic metabolites or the release of volatile organic compounds (VOCs) that directly inhibit herbivore growth or alter their behaviour and physiology (Clay and Schardl 2002; Rodriguez et al. 2009; Paul and Park 2013). For example, VOCs produced by *Cladosporium* spp. and *Trichoderma* spp. have been shown to deter insect herbivores and suppress their growth in various plant species (Davis et al. 2013; Contreras‐Cornejo et al. 2018). Endophytic fungi can also indirectly influence the secondary metabolite production in some plant species (Strobel and Daisy 2003; Zhang et al. 2006; Shrivastava et al. 2015; Bajaj et al. 2018). For instance, Shrivastava et al. (2015) reported that inoculating tomato plants with endophytic fungus enhanced insect resistance by increasing terpene accumulation and richness. While research on endophyte‐mediated resistance has advanced in annual plants, there is limited understanding of their role in conifers, particularly in relation to defences against insect herbivores (Barklund and Kowalski 1996; Miller 2011; Speed et al. 2015).

Defence metabolites play critical roles in tree resistance against insect herbivores in conifers (Phillips and Croteau 1999; Franceschi et al. 2005; Keeling and Bohlmann 2006; Celedon and Bohlmann 2019; Mageroy et al. 2023). The abundance and composition of these metabolites often vary within and between species (Moore et al. 2014; Raffa et al. 2017; Erbilgin 2019). This variation is often viewed as a result of co‐evolutionary feedback from their insect enemies (Moore et al. 2014; Ullah et al. 2021). More recently, the co‐evolutionary history between plants and their microbiome has been proposed as a crucial factor in modulating plant resistance to pests (Miller et al. 2008; Karst et al. 2015; Wang et al. 2019; Speed et al. 2015).

While the mechanism remains largely unexplored, fungal endophytes are thought to influence plant secondary metabolite synthesis through multiple pathways (Strobel and Daisy 2003; Verma et al. 2009; Hardoim et al. 2015). They may produce enzymes that modify the biosynthetic pathways in plants or share homologous genes involved in secondary metabolite production with hosts (Jennewein et al. 2001; Zhang et al. 2006; Rodriguez et al. 2009; Soliman and Raizada 2013). Additionally, shared biosynthetic pathways suggest that endophytes have acquired secondary metabolite production capabilities through co‐evolution with their hosts (Chandra 2012). These interactions highlight the importance of fungal endophytes in modulating conifer defence chemistry and enhancing resistance to herbivores.

We investigated the role of fungal endophytes in white spruce (*Picea glauca* (Moench) Voss) resistance to its primary insect defoliator, the eastern spruce budworm (ESB, *Choristoneura fumiferana* Clemens) (Lepidoptera: Tortricidae). We characterised the direct (i.e., fungal metabolites inhibit insect growth) and indirect (i.e., fungal endophytes alter tree defence metabolism) effects of fungal endophytes in white spruce defences. Understanding these two mechanisms is crucial for determining how endophytes contribute to host defence, as trees may benefit from multiple overlapping defences.

White spruce is the most widespread conifer species in the Canadian boreal forest (de Lafontaine et al. 2010). The ESB is the primary defoliator (foliage feeder) of white spruce, and during periodic outbreaks, repeated defoliations can kill millions of white spruce trees (Royama et al. 2017). White spruce foliage contains terpenoids and phenolics that affect the budworms, with both positive and negative impacts depending on their concentrations (Keeling and Bohlmann 2006; Delvas et al. 2011; Kumbasli and Bauce 2013; Mageroy et al. 2015; Parent et al. 2020; Ullah et al. 2024). These compounds vary across white spruce populations due to co‐evolution with pests and growing conditions (Delvas et al. 2011; Ullah et al. 2024).

White spruce foliage hosts a diverse community of endophytic fungi that may contribute to resistance to the ESB (Quiring et al. 2019). Earlier studies have shown that endophyte diversity varies among white spruce populations (Stefani and Bérubé 2006). These studies have reported that *Lophodermium piceae* is the most abundant species, followed by *Mycosphaerella* spp., *Hypoxylon* spp., and *Phomopsis* spp. (Koukol et al. 2012). Few studies have shown the direct effects of fungal endophytes on the ESB (Sumarah et al. 2005; Miller et al. 2008; Quiring et al. 2019, 2020). For example, Sumarah and Miller (2009) showed that *Phialocephala scopiformis* reduces feeding by the budworm and produces rugulosin, a secondary metabolite that inhibits budworm development. Similarly, Paul and Park (2013) identified several VOCs, including α‐pinene, (−)‐*trans*‐caryophyllene, tetrahydro‐2,2,5,5‐tetramethylfuran, many others from *Cladosporium* spp., but their effects on ESB are unknown. These findings demonstrate that fungal endophytes play a significant role in white spruce defence; however, a more comprehensive assessment of endophytic fungal diversity across white spruce populations is needed to clarify their broader impact on ESB. Furthermore, the composition and abundance of endophytic communities in white spruce are also influenced by plant genotype and environmental conditions (Stefani and Bérubé 2006); these factors could also shape white spruce resistance to herbivory (Rodriguez et al. 2009; et al. 2016a). Understanding these interactions is crucial for determining how endophytes contribute to host defence.

We proposed and tested a new Plant Partnership (PP) Hypothesis. This hypothesis has five predictions: (I) Endophytic fungi are widespread in white spruce, showing variations among genotypes and locations; (II) fungal endophytes produce secondary metabolites with anti‐herbivory properties; (III) consuming fungal endophytes or exposure to their VOCs adversely affect insect herbivores; (IV) the abundance of fungal endophytes is positively linked to the production of defence compounds; and thus (V) enhancing the abundance of fungal endophytes results in increased production of defence compounds. To test these predictions, we determined the composition of monoterpenes, sesquiterpenes, and endophytic fungal communities in 30 white spruce families across two sites in Alberta (Prediction I). We identified several endophytic fungal isolates (a single strain of a fungus maintained in pure culture) from white spruce foliage and characterised their secondary metabolites (II). We focused on monoterpenes and sesquiterpenes as fungal endophytes predominantly produce low molecular weight terpenes (Kim et al. 2004). We subsequently tested the effects of these isolates on the budworm performance by directly feeding the budworm on media amended with fungal mycelium or assessing the effect of their VOCs on the budworm feeding and attraction (III). Finally, we conducted a greenhouse experiment to demonstrate that changes in the fungal abundance in white spruce seedlings can alter their secondary metabolite production (IV) and that enhancing the fungal abundance in seedlings can increase their secondary metabolite production (V). In our investigation, we differentiated between the direct and indirect effects of fungal endophytes in white spruce resistance.

## 2 Materials and Methods

### 2.1 Field Site and Sample Collection

We collected current‐year and 1‐year‐old needles from the south aspects of 33‐year‐old white spruce trees (30 families, four trees/family) at two progeny sites, Carson Lake and Calling Lake (*n* = 240 samples per site) in Alberta in July 2020. Calling Lake (55°16′18.8″N, 113°09'54.6“W, 639 m elevation) has a clay loam soil type and annually experiences a mean annual temperature of 1.6°C with 467 mm precipitation. Trees were planted in May 1986. Carson Lake (54°23′37.8″N, 115°34′12.3″W, 1018 m elevation) has a slightly warmer mean annual temperature (2.9°C) and receives more precipitation (535 mm). Carson Lake also has clay loam soil, and trees were planted in May 1987. The research design remained consistent between the two sites. We sampled both needle types because ESB feeds on current‐year and older needles.

Foliar samples were packed in Ziploc bags and immediately transferred to a container containing dry ice in the field until subsequent storage in −40°C freezers in the laboratory. These samples were used for DNA sequencing, terpene analyses, and fungal isolation. We extracted the DNA separately from each needle type; however, our statistical analyses showed that since the needle type did not affect the terpene concentrations or the endophytic fungal abundance, we pooled the data from both needle types in each family in each site.

### 2.2 Identifying Foliar Endophytic Fungal Communities

We lyophilised needles at −50°C for 72 h in a freeze‐drier (Labconco Corp., Kansas City, MO, USA) and extracted DNA from 50 mg ground needle using an EZNA® HP Fungal DNA Kit with the concentration quantified using an ND‐1000 Nanodrop (NanoDrop, Wilmington, DE, USA). The fungal ITS regions were amplified using a two‐step PCR with fITS7 (Ihrmark et al. 2012) and ITS4 (White et al. 1990) primers and Illumina overhang adapters and sequenced on an Illumina Miseq (Illumina Inc., San Diego, CA, USA) platform. We included a detailed overview of DNA extraction and sequencing in Supporting Information Methods, *DNA extraction and sequencing*.

We conducted bioinformatics of Illumina paired‐end reads using the ‘DADA2’ pipeline in R software version 3.5.1 (R Core Team 2020). FastQC (Andrews 2010) was used to verify demultiplexed reads for non‐biological oligonucleotide content before importing them into “DADA2”. We used the ‘cutadapt’ plugin to preserve sequences from Illumina adapters and primer complements and used the ‘DADA2’ plugin (Callahan et al. 2016) to adjust read quality, filter chimeras, and resolve amplicon sequence variations (ASVs) of forward reads. We chose ASVs due to their higher resolution in defining fungal communities and the consistency in ecological results. Paired‐end readings were impossible because one of the two sequencing runs had poor reverse read quality. We used the UNITE dynamic classifier to assign taxonomy to ASVs, which evaluates individual lineages and assigns taxonomy on a 97%–98% confidence level (Abarenkov et al. 2010). We rarefied samples to 4320 sequences per sample for statistical analyses based on the rarefication curve. To categorise fungal guilds, we employed the FUNGuild database (Nguyen et al. 2016). We maintained a filtered table containing taxa assigned to the fungal endophytes, only including those with confidence rankings of “probable” or “highly probable”.

### 2.3 Monoterpene and Sesquiterpene Analyses

To characterise monoterpenes and sesquiterpenes, we extracted hexane‐soluble compounds from frozen ground needles (100 ± 5 mg fresh weight) (Ullah et al. 2021). We analyzed the samples using authentic standards in a Gas Chromatograph/Mass Spectrometer (GC/MS, Agilent 7890A/5062C, Agilent Tech., Santa Clara, CA, USA). The GC/MS settings for chemical analysis are detailed in the Supporting Information Methods, *Extraction of foliar terpenes*.

### 2.4 Endophytic Fungal Isolation

We surface sterilised needles by shaking them for 1 min in 70% ethanol, followed by 4 min in 3% sodium hypochlorite, and a final 1 min rinsed in double distilled water (Arnold et al. 2001). This process aimed to remove microbes from the needle surface. After sterilisation, we placed 10 needles (five from current and five 1‐year‐old) on the surface of Potato Dextrose Agar (PDA) media (BD Difco, Ann Arbor, MI, USA) in Petri Dishes (100 mm dia. × 15 mm ht.). We repeated these in each tree sampled. We did not track needles from individual trees, as we aimed to isolate the fungal endophytes at the family level. The fungi began to grow after 1 week, and we isolated the newly grown hyphae and transferred individual hyphae to new plates. We extracted DNA from individual isolates using Sigma Extraction Solution and Neutralisation Solution B according to manufacturer protocols (Sigma‐Aldrich; for details, see Supporting Information Methods, *Sanger sequencing and bioinformatics*). Many species of endophytic fungi do not grow on agar plates, and their presence can be more accurately determined through sequencing; consequently, the results from plating and sequencing vary.

The above process yielded 20 different endophytic fungal isolates, from which we selected 10 isolates based on their high abundance in Illumina sequencing or growth efficiency (Supporting Information S1: Table S1). Those isolates exhibiting very slow growth were excluded from further experiments.

### 2.5 Extraction of Fungal Metabolites From Cultured Isolates

To determine the metabolites (*volatiles and non‐volatiles*) of the 10 isolates, we grew them separately in 200 mL flasks containing Potato Dextrose Broth liquid media for 45 days; this yielded enough fungal mass for chemical analysis. The media was filtered using Whatman No. 1 filter paper in a vacuum‐connected 25 mm Büchner funnel. The resulting mycelium layer was transferred to a 10 mL glass tube (Kesell, Model No. BLP017) and dried in a freeze‐dryer (Labconco Corp.) for 72 h. We used 25 ± 5 mg (dry weight) of ground samples and extracted metabolites from them twice in 0.5 mL (50% ethyl acetate and 50% dichloromethane) with an internal standard of 0.004% pentadecane. We analyzed the samples using a GC/MS (Agilent Tech.). The GC/MS settings for these analyses are included in the Supporting Information Methods, *Extraction of fungal metabolites*.

### 2.6 VOC Collections From Fungal Isolates

To determine the VOCs of the isolates, we grew individual isolates on PDA by inoculating each Petri Dish separately from the advancing edge of an actively growing colony of each 9‐day‐old endophytic fungus. To identify fungal VOCs, we followed a push‐pull headspace method reported by Cale et al. (2016). Briefly, shortly after inoculation, two small Petri dishes (60 × 15 mm; Fisher Sci., Toronto, ON, CAN) with identical isolates were placed inside a volatile collecting chamber for 4 days. The chamber consisted of a 473 mL glass jar with Teflon tape on its threads and a metal cover. We attached the jar with a vacuum pump (Cole‐Parmer Canada Inc., Montreal, QC, CAN) and a flowmeter to maintain a constant airflow of 450 mL min^−1^ through the chamber lines. To filter and clean the air before it entered the collecting chamber, the intake channel was connected to a piece of Teflon tubing that was 30.5 cm long and filled halfway down with activated carbon (800 mg; 6–14 mesh; kept in place with glass wool). After 4 days, the headspace volatiles were then collected for 12 h in a 7.5 cm plastic tube containing activated carbon (150 mg; 6−14 mesh, Fisher Sci.) and secured with glass wool at both ends. After each collection, pumps were turned off; trap tubes were removed, wrapped in labelled aluminum foil, separately and kept at −40°C until chemical extraction. Each collection was replicated 10 times per isolate. For all VOC assays, we used sterile polystyrene Petri dishes. During fungal VOC collection, Petri dishes with or without PDA were employed to determine if any VOCs were produced from PDA and Petri dishes.

### 2.7 Testing the Effects of Fungal Endophytes on the ESB Feeding

We prepared three dosages of each isolate to test the effect of the mycelium of the 10 fungal isolates on budworms. We used the laboratory‐reared budworm larvae from the Insect Production and Quarantine Laboratories (Great Lakes Forestry Centre, Natural Resources Canada, Sault Ste. Marie, ON, CAN). Briefly, we amended the 100 mL McMorran diet (McMorran 1965) with an individual isolate separately by adding 50, 100, and 200 mg (*n* = 10). The mycelium was collected from liquid culture through filtration using Whatman No. 1 filter paper in a vacuum‐connected 25 mm Büchner funnel. We poured a 10 mL diet from the stock into a deli cup (60 mL) and placed one 4th instar pre‐weighed larva. We also prepared 110 cups of McMorran diet without isolates as controls. We weighed the diet before it was poured into the cups. We measured the larval weight again at the 6th instar. The duration from 4th to 6th instars was approximately 9–10 days. We recorded larval mortality, initial and final larval weight (mg), ingested food (g), and total larval development time from the 4th to 6th instars of each larva at the end of the experiment.

### 2.8 Testing the Effects of Fungal VOCs on the ESB Performance

To determine whether fungal VOCs affected the growth of budworms, we grew the 10 isolates on PDA as described above. Immediately following fungal inoculation, two plates without lids were placed at the bottom of a glass jar. To facilitate the accumulation of fungal‐derived VOCs, we capped the jars. We hung a deli cup containing the McMorran diet and one 4th instar larva above the plates, allowing a 5 cm gap. To facilitate the transmission of fungal volatiles, we created 10 openings (each 2 mm in dia.) on the top and bottom surfaces of the cup. Each isolate had 10 replicates. The control group consisted of the 10 jars prepared with media plates without the fungus. We measured the larval weight at the 4th and 6th instars.

### 2.9 Testing the Effects of Endophytic Fungal VOCs on the Budworm Larval Choice

In an olfactometer, the behavioural responses of budworm larvae to fungal VOCs were studied. We prepared PDA media plates and separately inoculated them with single isolates. The 10 isolates were grown on media for 4 days. We modified the olfactometer body with a 15‐cm piece of flexible polyvinyl chloride tube measuring 12 mm OD, 8 mm ID, and 1 mm thickness as the body of the olfactometer (Choe et al. 2016). Briefly, we placed three pieces (4 mm each) of 4‐day‐old fungal inoculum and three pieces (4 mm each) of McMorran diet into a 2 mL glass vial (Agilent Tech.). This vial was connected to one end of a tubing system. On the other end, we attached a separate glass vial containing only PDA media and McMorran diet of the same size, which served as the control. A single 4th instar larva was introduced into the tubing by making a 2 mm slit cut at the centre of the tubing. After 1 h, we recorded the larval position, either near treatment or control. Ten larvae were examined for each isolate.

## 3 Testing Whether the Changes in the Fungal Endophyte Communities Affect the Secondary Metabolites of White Spruce Seedlings

### 3.1 Fungal Spore Preparation

We selected the most dominant five species of the 10 fungal isolates. We prepared PDA plates for each species and incubated them at 23°C for 24 h before fungal inoculation. We inoculated the 9‐day‐old culture from the advancing edge of an actively growing fungus colony; one fungal species was inoculated per dish. To achieve enough fungal spore production, 25–30 plates were inoculated with individual fungi. We collected fungal spores by gently rubbing the surface of the fungal culture with distilled water after 10–14 days when the culture had produced spores. Using these spores, we prepared the spore stock suspension for each fungus using an established protocol (Raghavendra and Newcombe 2013). Briefly, spore suspensions were prepared using fungal cultures varying in age from 10 to 14 days. Multiple culture plates (15–20) from each species were used to make a homogenised spore suspension in sterile distilled water. The spore concentrations were 1 × 10^8^ CFU (Colony Forming Unit) mL^−1^. The spore suspension was immediately applied following preparation.

### 3.2 Fungal Application on White Spruce Seedlings

We grew 20 seedlings from seeds of each of 30 white spruce families from the progeny trials in a growth chamber under controlled conditions (23°C/5°C day/night, with a 16 h–8 h light‐dark cycle) for 10 months (a total of 600). Seedlings underwent a dormancy period (see details on seedling dormancy and fertilisation protocol in Supporting Information Methods, *Seedling fertilisation and dormancy protocol*). After dormancy, we randomly divided seedlings from each family into two groups (*n* = 10 per treatment per family). One group was inoculated with a mixture of five species of fungal endophytes by leaf‐spraying. The five fungal spore suspensions were mixed with Tween 20 (1 mL/L) for inoculation, with the spores from each fungal species combined in equal ratios to create a uniform mixture. Each seedling received 10 mL of this solution via a sterilised hand sprayer. The second group was sprayed only with Tween 20 suspension.

After inoculation, the seedlings were covered with a plastic bag for 24 h to maintain the high humidity inside the bags and accelerate fungal infection. After 2 months, we collected the needles from each seedling and stored them at −40°C until processing. We separately weighed the fresh biomass of needles and above‐ground woody tissues without needles. We used the abovementioned methods to identify the fungal endophyte abundance and monoterpene and sesquiterpene concentrations. Foliar fungal sequence data from white spruce seedlings was deposited in the NCBI (BioProject ID: PRJNA954773).

### 3.3 Data Analyses

We first tested whether terpene concentrations and endophytic fungal abundance varied among families by needle type (current‐year vs. 1‐year). As needle type did not affect terpenes nor fungal abundance, we pooled data from both needle types in each family in each site.

Since read counts obtained through metabarcoding exhibit a strong correlation with the actual read abundances measured using droplet digital PCR assays in the samples (Wang et al. 2024), we incorporated the read abundance data into our analyses. We calculated the proportion of fungal guilds and endophytic fungal genera from each site. We checked the data for the assumptions of homoscedasticity and normality by using Levene's and Shapiro–Wilk tests, respectively. Where necessary, we transformed (log + 1) data before analysis. We tested the effect of sites and families on the total terpenes and endophytic fungal abundance for statistical significance by ANOVA and *t*‐test, followed by post hoc pair‐wise differences using Tukey's HSD test. We did not find significant interaction between site and family on the total terpenes and endophytic fungal abundance; hence, we did not report these results. We conducted linear regression analysis between the total monoterpenes and sesquiterpenes relationship. We generated heat maps to visualise the fungal metabolites and VOCs of the 10 fungal isolates. We conducted an NMDS analysis to visualise the spread and relationship among fungal metabolites and VOCs on different fungi. We conducted a one‐way ANOVA to determine larval weight change in response to the fungal mycelium and VOCs compared to the control. A two‐sample *t*‐test was performed on the data obtained from the olfactometer experiment.

We tested the impact of fungal inoculations on the total monoterpenes, sesquiterpenes, and endophytic fungal abundance of seedlings for statistical significance by a two‐sample *t*‐test. We used PERMANOVA to determine the differences between individual seedling foliar terpenes and endophytic fungal abundance in inoculated and control treatments. Moreover, we used CAP ordination techniques to explore the variables visually. We also conducted a regression analysis to determine the correlation between total terpenes, sesquiterpenes, and endophytic fungal abundance using seedlings in inoculated and control groups. We considered significance at *α* = 0.05. Statistical software R v3.4.4 was used for all statistical analyses.

## 4 Results

### 4.1 Foliage Fungal Community Composition

**Figure 1:**
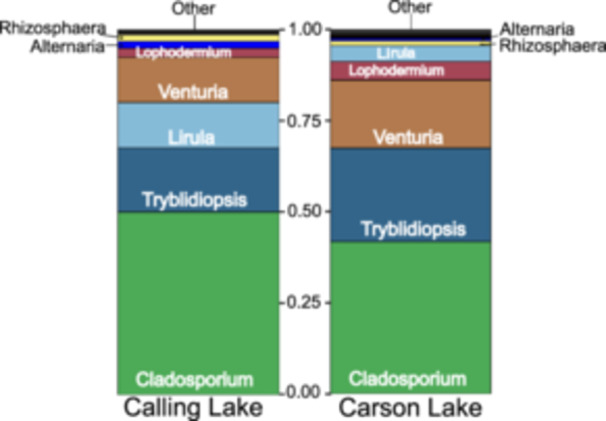
Proportions of endophytic fungal genera identified in *Picea glauca* families from Calling Lake and Carson Lake, Alberta (Canada) based on the FUNGuild database. Other in **Carson lake**: Agrocybe; Cadophora; Chalara; Coccomyces; Comoclathris; Cryptosphaeria; Cytospora; Diaporthe; Didymella; Exidia; Geopyxis; Gnomoniopsis; Hypoderma; Johnalcornia; Kabatiella; Kalmusia; Leptodontidium; Linospora; Lophodermella; Melanconis; Neptunomyces; Ophiognomonia; Paecilomyces; Paraconiothyrium; Paramassariosphaeria; Periconia; Pezizales; Phialocephala; Plagiostoma; Pleurotus; Pseudopithomyces; Pyrenophora; Rachicladosporium; Rhytisma; Stempodiella; Stemphylium; Toxicocladosporium; Valsalnicola. Other in **Calling Lake**: Celosporium; Chalara; Cadophora; Coccomyces; Comoclathris; Cryptosphaeria; Endoconidioma; Exidia, Geopyxis; Kabatiella; Melanconis; Neptunomyces; Ophiognomonia; Paecilomyces; Paraconiothyrium; Paramassariosphaeria; Periconia; Phialocephala; Plagiostoma; Pleurotus; Pseudopithomyces; Pyrenophora; Rachicladosporium; Rhytisma; Stemphylium; Ulocladium.

We sequenced 18.3 million DNA reads from two Illumina MiSeq runs, averaging 38 936 reads per sample. After applying ‘DADA2’ quality control and filtering, 13.1 million reads, representing 12 681 ASVs, remained for analysis, corresponding to 1198 taxa. We subsampled 4580 sequences per sample for comparability based on species rarefaction curves. Among 11 fungal guilds identified, Carson Lake had a higher proportion of endophytes than Calling Lake (Supporting Information S1: Figure S1). Since we focused on endophytic fungi, we excluded other fungal guilds from subsequent analysis. The five most abundant endophytic fungal genera, *Cladosporium*, *Tryblidiopsis*, *Venturia*, *Lophodermium*, and *Lirula*, were consistent, but their abundance varied between sites: *Tryblidiopsis*, *Venturia*, and *Lophedermium* were most abundant in Carson Lake, while *Cladosporium* and *Lirula* were in Calling Lake. Carson Lake contained 45 endophytic genera, whereas Calling Lake had 33 (Figure 1). Carson Lake also had higher fungal abundance, with 815 ± 49 reads compared to 606 ± 31 reads in Calling Lake (*t*_469_ = −3.51, *p* < 0.001). Fungal abundance varied among families (Supporting Information S1: Figure S2). Additional results were included in Supporting Information results, *Foliage fungal community composition*.

### 4.2 Terpene Composition and Concentrations Across Sites and White Spruce Families

We identified 12 monoterpenes and nine sesquiterpenes at both sites (Supporting Information S1: Tables S2–S5 and Figures S3–S6). Calling Lake had 1.53× more total monoterpene concentration than Carson Lake (*t*_469_ = 6.841, *p* < 0.001). In contrast, Carson Lake had 1.74× more total sesquiterpenes than Calling Lake (*t*_469_ = −3.867, *p* < 0.001). The individual and total monoterpene and sesquiterpene concentrations varied among families in each site (Supporting Information S1: Figures S3–S7; Supporting Information Results, *Terpene composition and variations across sites and spruce families*).

### 4.3 Sanger Sequencing Results From Cultured Isolates

Using Sanger sequencing, we identified foliar fungal endophytes from mature trees, including both current and 1‐year‐old needles. We isolated 30 fungal cultures from both sites and successfully amplified 25 of them. Of the 25 successful amplifications submitted, 21 yielded informative sequences, and sequence clustering resulted in 21 OTUs, which matched sequences accessioned in GenBank. Among these, 20 OTUs were identified as foliar fungal endophytes and one as an ectomycorrhizal fungus and showed the best matches in the FUNGuild database. Among 20 fungal endophytes, one OUT was duplicated. The 10 fungal endophytes used in our experiments showed query coverage and identity percentage from 96% to 100% (Supporting Information S1: Table S1).

The number of fungal endophytes varied between the culture‐based method and Illumina sequencing (DNA‐based method). We identified 20 fungal endophytes from Sanger sequencing; however, Illumina sequencing provided 45 fungal endophyte genera. This discrepancy was anticipated, as not all fungal endophytes are culturable, which was demonstrated in our study using white spruce needles on PDA from all the families tested. Fungal endophytes may require specific nutrients or symbiotic relationships with the host that artificial media cannot replicate.

### 4.4 Metabolic Profiles of 10 Endophytic Fungal Isolates

**Figure 2:**
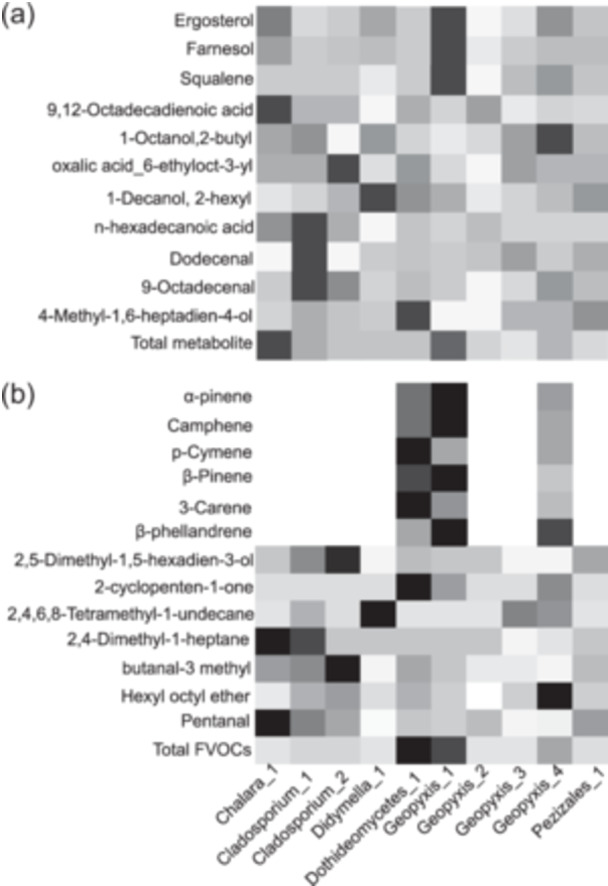
A heatmap showing (a) the metabolite profiles of 10 endophytic fungi and (b) the volatile organic compounds profiles of 10 endophytic fungi. The lowest to highest concentrations (ng mg^−1^ dry weight) were demonstrated by light to dark colours, respectively. The concentration of individual compounds was compared across endophytic fungi.

We identified 11 metabolites across 10 isolates of fungal mycelium, including one sterol, one oxygenated monoterpene, one sesquiterpene, one triterpene, two fatty acids, three fatty aldehydes, and two glycols (Supporting Information S1: Table S6). We generated a heat map based on metabolite concentrations for each isolate (Figure 2a, Supporting Information S1: Table S7). *Chalara*_1 had the highest total concentration, followed by *Geopyxis*_1, which also showed higher concentrations of ergosterol, farnesol, and squalene than other isolates. *Dothideomycetes*_1 produced a higher concentration of 4‐methyl‐1,6‐heptadien‐4‐ol, while *Cladosporium*_1 had higher concentrations of n‐hexadecanoic acid, dodecanal, and 9‐octadecenal. NMDS analysis coupled with PERMANOVA showed a wide dispersion among isolates with respect to their metabolite composition (Supporting Information S1: Figure S8a). Notably, ergosterol, farnesol, and triterpene squalene were correlated with *Geopyxis*_1. The metabolite concentrations also varied across isolates (*R* ^2^ = 0.13, *F*_9_ = 27.31, *p* < 0.001).

### 4.5 Effects of Endophytic Fungal Mycelia on the Budworm Weight

**Figure 3:**
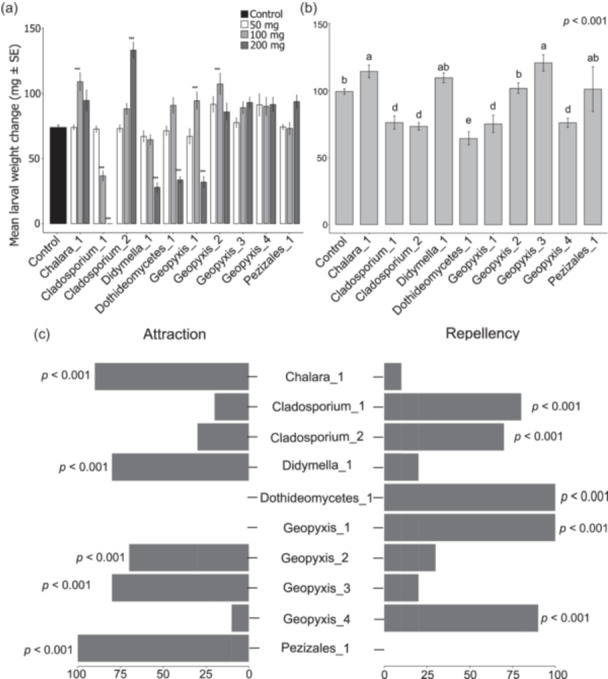
Mean (±SE) larval weight change (mg) of *Choristoneura fumiferana* on (a) 10 endophytic fungi with three different dosages and (b) endophytic fungal volatile organic compounds. (a) The one‐way ANOVA was conducted separately by comparing the control versus each fungus with three dosages separately. The asterisk shows a significant difference from the control. (b) One‐way ANOVA was conducted among different fungi and control treatments. Bars with different letters are statistically different (Tukey HSD tests, *p* < 0.001). (c) Percent attraction and repellency of spruce budworm larvae to endophytic fungi. The larval response to the fungal treatments is shown in the right bar graph, whereas the control group's response is depicted in the left bar graph. Two‐sample *t*‐test results demonstrate a statistically significant difference at *p* < 0.05.

The fungal treatments had varying effects on larval weight, depending on the isolates and the doses applied (Figure 3a). *Cladosporium*_1 at 200 mg dose resulted in 100% mortality. In contrast, lower doses reduced the larval weight without causing mortality. *Didymella*_1, *Geopyxis*_1, and *Dothideomycetes*_1 at the highest dose reduced larval weight relative to the control. In contrast, *Clasosporium*_2 at 200 mg increased larval weight, with no effects at lower doses.

### 4.6 VOC Profiles of 10 Fungal Isolates

We characterised 13 fungal VOCs from five classes, including seven monoterpenes, two aldehydes, two alkanes, one ether, and one ketone (Figure 2b, Supporting Information S1: Table S8). *Dothideomycetes*_1 had the highest total VOC concentration, followed by *Geopyxis*_1 (Supporting Information S1: Table S9). Only *Dothideomycetes*_1, *Geopyxis*_1, and *Geopyxis*_4 produced monoterpenes, and one oxygenated monoterpene was also detected (Supporting Information S1: Tables S8 and S9). Following NMDS analysis and PERMANOVA, correlations among fungal VOCs and isolates *Geopyxis*_1, *Geopyxis*_4, and *Dothideomycetes*_1 were identified (Supporting Information S1: Figure S8b). Overall, the amounts of fungal VOCs differed substantially amongst isolates (*R* ^2^ = 0.23, *F*_9_ = 17.44, *p* < 0.001).

### 4.7 Effects of Endophytic Fungal VOCs on the Budworm Performance

Fungal VOCs also affected the larval weight (Figure 3b). *Geopyxis*_1, *Geopyxis*_4, *Cladosporium*_1, *Cladosporium*_2, and *Dothideomycetes*_1 reduced larval weight, whereas, *Geopyxis*_3 and *Chalara*_1 improved it relative to the control. The remaining three isolates had no effects. We assessed the attraction of larvae to fungal VOCs in olfactometer assays. A two‐sample *t*‐test showed differences between the control and fungal treatments (Figure 3c). *Geopyxis*_1 and *Dothideomycetes*_1 showed 100% repellency, while *Geopyxis*_4, *Cladosporium*_1, and *Cladosporium*_2 exhibited 90%, 80%, and 70% repellency, respectively. The remaining five isolates were attractive to the budworm.

### 4.8 Foliage Fungal Community of White Spruce Seedlings

**Figure 4:**
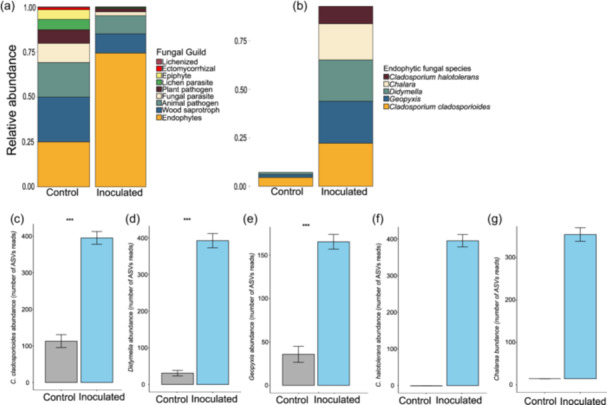
Relative abundance of (a) fungal guilds, and (b) foliar endophytic fungal species identified in *Picea glauca* seedlings following control and inoculation based on FUNGuild database (Nguyen et al. 2016). (c–g) show mean (±SE) endophytic fungal read abundance of (c) *Cladosporium cladosporioides*, (d) *Didymella*, (e) *Geopyxis*, (f) *Cladosporium halotolerans*, and (g) *Chalara*. The two‐sample *t*‐test was conducted for comparing control and inoculated seedling groups. Asterisks show significant differences from the control seedlings at *p* < 0.01. [Color figure can be viewed at wileyonlinelibrary.com]

**Figure 5:**
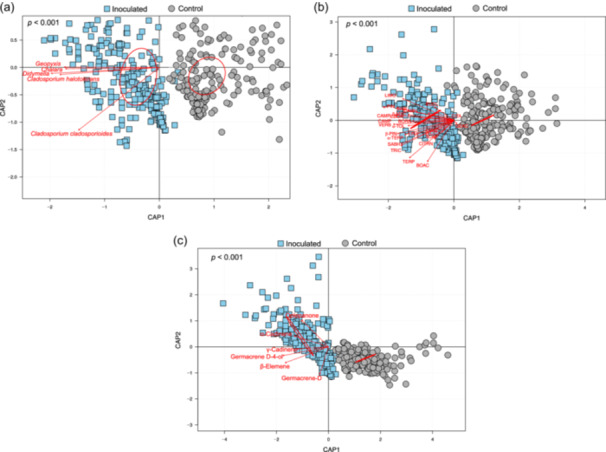
Canonical Analysis of Principal Coordinates (CAP) plot the distribution of (a) individual endophytic fungal abundance, (b) individual monoterpenes, and (c) individual sesquiterpenes in inoculated and control seedlings of *Picea glauca*. Red vectors represent (a) individual fungal endophytes, (b) individual monoterpenes, and (c) individual sesquiterpenes. PermANOVA was used for statistical analyses (*p* < 0.001). Abbreviations for monoterpenes: BPIN = β‐pinene, APIN = α‐pinene, LIMO = limonene, EUCL = eucalyptol, SAB = sabinene hydrate, β‐MYC = β‐myrcene, CAPR, camphor, CAMP = camphene, 3CAR = 3‐carene, TERP = terpinolene, CAMR = camphor, α‐TOL = α‐terpineol, α‐TERP = α‐Terpinene, TERP = γ‐Terpinene, BORN = borneol, EBOR = endo‐borneol, LINA = linalool, BOAC = bornyl acetate, GEAC = geranyl acetate, β‐CIT = β‐citral, β‐PHE = β‐phellandrene, VERB = verbenone, TRIC = tricyclene, CITRN = citronellol. [Color figure can be viewed at wileyonlinelibrary.com]

Both inoculated and control seedlings exhibited nine fungal guilds, but the relative abundance of endophytes in the inoculated seedlings was nearly three times of the control (Figure 4a). This increase paralleled to a decrease in the relative abundance of the other eight guilds. Seedlings showed an overall ~0.90 incidence proportion, which was ninefold higher than the control. Among the inoculated fungi, *Cladosporium cladosporioides* had the highest proportion, followed by *Geopyxis* sp., *Didymella* sp., and *Chalara* sp., while *C. halotolerans* had the lowest (Figure 4b). In contrast, control seedlings had only *C. cladosporioides, Geopyxis* sp., and *Didymella* sp., with an overall incidence proportion of around 0.10, and lacked *C. halotolerans* and *Chalara sp*., confirming successful inoculation. Comparisons of fungal species between inoculated and control seedlings showed higher levels in the former (Figure 4c–g). The CAP and PERMANOVA analyses indicated a significant increase in the abundance of five fungal endophytes in inoculated seedlings (Figure 5a). *Cladosporium halotolerans*, *Didymella* sp., *Geopyxis* sp., and *Chalara* sp. clustered closely, while *C. cladosporioides* was distinct from the others.

### 4.9 Endophytic Fungal Abundance in White Spruce Seedlings

We examined the impact of fungal inoculation across white spruce families by comparing inoculated and control groups. Overall, fungal inoculation led to significant increases in fungal abundance, though the levels varied by species and family (Supporting Information S1: Figure S9). *Cladosporium cladosporioides* was more abundant in inoculated families, except for two, with mean read abundances ranging from 350 to 570, while control families exhibited a much lower range between 50 and 180. Family 203 had the highest abundance, whereas families 133, 156, and 157 had the lowest. In the control group, *C. cladosporioides* was absent in only one family, while the others had an average read abundance below 200. Similarly, *Didymella* sp. showed significantly higher abundance in inoculated families, with mean read counts ranging from 125 to 800, whereas control families had counts below 100. Five control families showed no evidence of *Didymella* sp. Among the inoculated families, family 148 had the highest abundance, while families 128, 157, and 158 had the lowest. For *Geopyxis* sp., all but one inoculated family (133) exhibited substantially higher levels than the controls. The mean read abundance in inoculated families ranged from 40 to 370, while it remained below 100 in control families. None of the six control families showed any signs of *Geopyxis* sp. presence. Family 148 had the highest abundance, whereas families 128 and 133 had the lowest. Neither *C. halotolerans* nor *Chalara* sp. were present in any control families. However, among inoculated families, *C. halotolerans* had mean read counts ranging from 80 to 580, while *Chalara* sp. showed a wide abundance variation ranging from 18 to 510 reads.

### 4.10 Foliage Terpene Composition of White Spruce Seedlings

Post‐inoculation, total monoterpenes (control vs. inoculated: 5400 ± 205 vs. 8000 ± 260 ng mg^−1^) and sesquiterpenes (175 ± 9 vs. 240 ± 11 ng mg^−1^) increased by 33% and 28%, respectively, in inoculated seedlings (Supporting Information S1: Tables S10 and S11). The CAP analysis revealed a skewed distribution of monoterpenes towards inoculated seedlings (Figure 5b), and PERMANOVA indicated significantly higher concentrations of individual monoterpenes in these seedlings. Geranyl acetate was the exception, found only in control seedlings. The CAP analysis also showed distinct clustering of sesquiterpenes in inoculated seedlings (Figure 5c), with PERMANOVA confirming higher concentrations of all sesquiterpenes in the inoculated group.

Total monoterpene and sesquiterpene concentrations varied among control and inoculated families (Supporting Information S1: Figure S10). In total, 23 inoculated families exhibited higher monoterpene concentrations, ranging from 4850 to 11 850 ng mg^−1^, while the control group ranged from 1500 to 7900 ng mg^−1^, corresponding to a 40%–66% increase overall. Seven families showed no difference from the control (Supporting Information S1: Figure S10a). Seventeen inoculated families demonstrated higher sesquiterpene concentrations, ranging from 120 to 420 ng mg^−1^, compared to 80 to 250 ng mg^−1^ in the control group, corresponding to a 37%–40% increase overall. One control family (1980) had higher sesquiterpene concentrations than the inoculated group, while the remaining 12 inoculated families showed similar concentrations (Supporting Information S1: Figure S10b).

### 4.11 Relationship Between Endophytic Fungal Abundance and Terpenes in White Spruce Seedlings

**Figure 6:**
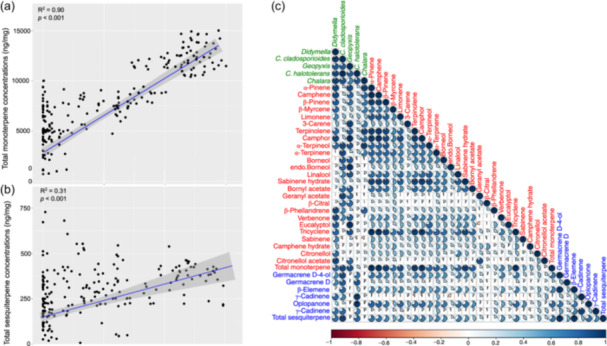
The relationship between total fungal endophytes, (a) total monoterpene concentrations (ng mg^−1^), (b) total sesquiterpene concentrations (ng mg^−1^) of *Picea glauca* seedling foliage, line and shad denote regression line and 95% confidence interval, respectively and (c) Pearson correlation pie charts (r) for individual fungal endophytes (green text), monoterpenes (red text), and sesquiterpenes (blue text) of *P. glauca* seedlings. The darker the blue or red pie charts, the closer the *r* value is to 1 or −1. [Color figure can be viewed at wileyonlinelibrary.com]

We conducted a linear regression analysis to examine the correlation between total endophytic abundance and the concentrations of monoterpenes and sesquiterpenes of seedlings in both inoculated and control groups. Different families with varying endophytic fungal abundance demonstrated a positive relationship with overall monoterpene and sesquiterpene concentrations (Figure 6a,b). Pearson correlation analysis indicated that fungal endophytes correlate with individual terpene concentrations (Figure 6c). Except for *C. halotolerans*, all four fungal endophytes correlated with most individual and total monoterpenes. *Didymella* sp., *C. cladosporioides*, and *C. halotolerans* were the only fungal endophytes positively correlated with individual and total sesquiterpenes. Our data suggest that an increase in total endophytic abundance corresponds to higher monoterpene and sesquiterpene concentrations.

## 5 Discussion

Our investigations into tripartite interactions have enhanced our understanding of the importance of fungal endophytes in the white spruce‐ESB system. We found considerable variation in fungal endophyte abundance across white spruce families, within and between sites, identifying up to 45 endophytic fungal genera. Terpene concentrations also varied among white spruce families and between sites. Moreover, we identified 11 secondary metabolites directly from fungal mycelium and 13 fungal VOCs through headspace analysis. Our bioassays showed direct anti‐herbivore resistance to the budworm, attributed to the toxicity of fungal mycelium or their VOCs. Inoculation experiments with seedlings further showed that shifts in fungal endophyte communities can influence terpene concentrations in foliage. We found a strong positive relationship between total monoterpene or sesquiterpene concentrations and fungal abundance, suggesting a possible influence of endophytic fungi in terpene production. Overall, these results align with the predictions of the PP Hypothesis, supporting the co‐evolutionary relationships between fungal endophytes, defence compounds, and their effects on budworm herbivory.

### 5.1 The Influence of Tree Families on Endophyte Fungal Communities and Abundance

The five predominant endophytic fungal genera were *Cladosporium*, *Tryblidiopsis*, *Venturia*, *Lophodermium*, and *Lirula*. Some of these genera were also reported in white spruce in other regions and other conifer species. For instance, *Cladosporium* and *Lophodermium piceae* were identified in the foliage of white spruce in southern Québec (Stefani and Bérubé 2006). Similarly, *Cladosporium* was the most abundant genus among the four *Pinus* species in Korea (Rim et al. 2021). In Calling Lake, we observed variations in the overall fungal abundance, with different white spruce families suggesting genotypic specificity, supporting the results of earlier studies which have shown that tree genotype can influence the fungal endophyte abundance in conifer species (Wang and Guo 2007; Rajala et al. 2014). Together, these findings suggest a close association between plant genotypes and endophytic fungal abundance (Bálint et al. 2013).

### 5.2 Fungal Endophytes as Crucial Partners in Enhancing White Spruce Resistance to ESB

Fungal endophytes provided direct anti‐herbivore resistance to the budworm, as demonstrated by the direct toxicity of fungal mycelium or their VOCs. In feeding bioassay, fungal mycelia induced larval mortality of up to 100% at higher doses, while lower doses substantially reduced larval fitness, agreeing with the results of earlier studies on direct anti‐herbivory effects of fungal mycelium (Sumarah and Miller 2009; Quiring et al. 2019). Additionally, VOCs from the five fungal species reduced the budworm weight. A reduction in the budworm defoliation and survival was similarly reported after inoculating white spruce trees with the endophytic fungus *Phialocephala scopiformis* in Eastern Canada (Quiring et al. 2020). Furthermore, fungal VOCs repelled the budworm larvae, further contributing to the white spruce defences. A similar phenomenon is also observed in other plant‐endophyte‐pest systems. For instance, the endophyte *Muscodor vitigenus* in liana plants, *Paullina paullinioides*, produced naphthalene that repelled the sawfly, *Cephus cinctus* (Daisy et al. 2002). Likewise, an endophyte strain (AP‐796) obtained from green foxtail (*Setaria viridis*) produced 3‐(4‐methylfuran‐3‐yl)‐propan‐1‐ol, that deterred the stink bugs, *Eysarcoris ventral* (Nakajima et al. 2010). These results highlight the ecological significance of interactions between plant endophytes and insect herbivores.

In our study, we could not quantify fungal concentrations in individual needles due to methodological limitations. To address this, we conducted a dose‐response assay to evaluate how endophytes influence budworm feeding behaviour. This assay showed that the budworm response to fungal VOCs was concentration‐dependent. Specifically, endophytes with higher VOC concentrations (33–102 ng mg^−1^) repelled the budworm, while lower concentrations (21–26 ng mg^−1^) either attracted them or had no effect. This suggests that fungal VOCs may serve a dual role—affecting larval development and feeding preferences—which could reduce herbivory pressure and enhance tree resistance to the budworms. While larval movement is limited between trees, VOC‐mediated deterrence remains ecologically relevant. The 10 endophytes used in the VOC analysis may not truly reflect the diverse endophyte community in white spruce foliage, potentially leading to different outcomes in nature. Despite these limitations, our findings offer valuable insights into white spruce defence mechanisms.

### 5.3 Fungal Endophytes Influence the Production of White Spruce Defence Metabolites

Fungal endophytes influence white spruce defence metabolites, as demonstrated by a key finding in our study. Inoculating white spruce seedlings with a blend of five foliar endophytic fungi increased terpene production, with 76% and 56% of inoculated seedlings having higher concentrations of monoterpenes and sesquiterpenes, respectively, compared to non‐inoculated controls. Notably, some families (e.g., 128, 180, and 1918) showed several‐fold increases in total monoterpene concentrations, while similar increases in total sesquiterpene concentrations were observed in several families (e.g., 128, 157, and 180). These findings align with those of Sumarah et al. (2005), who reported that inoculating white spruce seedlings with the rugulosin‐producing endophyte 5WS22E1 (DAOM 229536), led to elevated concentrations of rugulosin, a cyclic ketone and organic polycyclic secondary metabolite. Furthermore, in the current study, fungal endophytes produced five different classes of secondary metabolites, including several monoterpenes abundant in white spruce foliage (Ullah et al. 2024). A preliminary estimate suggests that the fungal terpene 3‐carene is approximately seven times more concentrated in the mycelium (22 ng mg^−1^) than in needles (3 ng mg^−1^), highlighting the potential role of fungal endophytes in modulating host terpene concentrations. However, it remains unclear whether white spruce foliage contains any other classes of secondary metabolites produced by these endophytes. Collectively, these studies highlight the role of fungal endophytes in enhancing the host's metabolic diversity and abundance, including metabolites relevant to defence (Strobel and Daisy 2003; Miller et al. 2008; Ullah et al. 2024).

Although the specific mechanisms underlying fungal endophyte‐induced terpene production were not explored in this study, existing research suggests multiple pathways through which endophytes may influence host secondary metabolism. One pathway is that endophytes modulate terpene biosynthesis by altering gene expression in the host, either through direct enzymatic activity or signalling interactions that activate terpene biosynthetic pathways (Zhang et al. 2006; Rodriguez et al. 2009). Additionally, homologous genes for secondary metabolite production shared between endophytes and their hosts may facilitate metabolic crosstalk, potentially leading to modified metabolite profiles (Jennewein et al. 2001; Kusari and Spiteller 2011; Jia et al. 2016). This phenomenon has been observed in other plant‐endophyte systems, such as the taxol‐producing *Paraconiothyrium* sp., which upregulated taxol biosynthetic genes in *Taxus* (Soliman and Raizada 2013). Given that endophytes and host plants often share biosynthetic pathways and enzymatic machinery, it is likely that fungal endophytes have evolved the ability to influence host secondary metabolism through long‐term co‐evolutionary interactions (Chandra 2012). Understanding these interactions in white spruce could provide valuable insights into the broader ecological implications of endophyte‐mediated plant defence strategies.

### 5.4 Conclusion

Our study validates the PP Hypothesis, demonstrating that fungal endophytes are cryptic partners in enhancing white spruce resistance to the ESB. These endophytes provide direct anti‐herbivore effects through toxic fungal mycelium and their VOCs. Our study links fungal abundance to the production of defence metabolites, emphasising the influence of endophytes on terpene synthesis and richness. Such richness is critical for natural selection, supporting optimal phenotypes with effective herbivory resistance through synergistic interactions among metabolites (Richards et al. 2015). Nevertheless, the effect of endophytes on insect herbivory is likely more complex than presented here. For example, the fungi‐produced secondary metabolites can lower the diversity of insects feeding on plants (Jaber and Vidal 2010) and impede the insects’ ability to obtain plant nutrition (Contreras‐Cornejo et al. 2018).

## Conflicts of Interest

The authors declare no conflicts of interest.

## Supporting information


**Figure S1.** Proportions of fungal guilds identified in *Picea glauca* families from Calling Lake and Carson Lake, Alberta (Canada) based on the FUNGuild database (Nguyen *et al*. 2016).
**Figure S2.** Mean (±SE) endophytic fungal abundance (number of reads) observed among 30 *Picea glauca* phenotypes in **(a)** Calling Lake and **(b)** Carson Lake. Bars with different letters are statistically different (Tukey HSD tests). *P*‐value indicates the results of one‐way ANOVA. Calling Lake (a): F_29,210_=2.95, *P* < 0.001, n=4/family; Carson Lake (b): F_29,197_=1.99, *P* < 0.001, n=4/family.
**Figure S3.** A heatmap showing the monoterpenes profiles of 30 families of *Picea glauca* that were sampled from Calling Lake, Alberta (Canada). The lowest to highest concentrations (ng mg^−1^ Fresh Weight) were demonstrated by light to dark colours, respectively. Concentrations of individual compounds were compared across families.
**Figure S4.** A heatmap showing the monoterpenes profiles of 30 families of *Picea glauca* that were sampled from Carson Lake, Alberta (Canada). The lowest to highest concentrations (ng mg^−1^ Fresh Weight) were demonstrated by light to dark colours, respectively. Concentrations of individual compounds were compared across families.
**Figure S5.** A heatmap showing the sesquiterpenes profiles of 30 families of *Picea glauca* that were sampled from Calling Lake, Alberta (Canada). The lowest to highest concentrations (ng g^−1^ Fresh Weight) were demonstrated by light to dark colours, respectively. Concentrations of individual compounds were compared across families.
**Figure S6.** A heatmap showing the sesquiterpenes profiles of 30 families of *Picea glauca* that were sampled from Carson Lake, Alberta (Canada). The lowest to highest concentrations (ng mg^−1^ Fresh Weight) were demonstrated by light to dark colours, respectively. Concentrations of individual compounds were compared across families.
**Figure S7.** Mean concentrations (±SE) of total monoterpenes and sesquiterpenes from different *Picea glauca* phenotypes in **(a)** Calling Lake and **(b)** Carson Lake. Bars with different capital letters representing total monoterpenes and small letters representing total sesquiterpenes are statistically different (Tukey HSD tests). *P*‐values indicate the results of one‐way ANOVA. Calling Lake: Monoterpenes: F_29,210_=2.3, *P*< 0.001; Sesquiterpenes: F_29,210_=3.94, *P*< 0.001. Carson Lake: Monoterpenes: F_29,210_=3.99, *P*< 0.001; Sesquiterpenes: F_29,210_=4.63, *P*< 0.001.
**Figure S8 (a)** An NMDS showing the distribution of fungal metabolites (red vectors) from 10 endophytic fungi (black text). **(b)** An NMDS plots the distribution of individual endophytic fungal volatile organic compounds abundance (red vectors) in different endophytic fungi (black text).
**Figure S9.** Mean (±SE) endophytic fungal read abundance of **(a)** *Cladosporium cladosporioides*, **(b)** *Didymella*, **(c)** *Geopyxis*, **(d)** *Cladosporium halotolerans*, and **(e)** *Chalara* among seedlings of 30 *Picea glauca* families. The two‐sample t‐test was conducted for individual comparisons of control and inoculated families. Asterisks show significant differences from the control seedlings at *p* < 0.01.
**Figure S10.** Mean concentrations (±SE) of **(a)** total monoterpenes and **(b)** total sesquiterpenes among families of *Picea glauca* seedlings. The two‐sample t‐test was conducted for individual comparisons of control and inoculated seedling families. Asterisks show significant differences from the control seedlings at *p* < 0.01.
**Table S1.** Operational taxonomic units (OTUs) of selected endophytic fungi found on *Picea glauca* foliage collected in Calling Lake and Carson Lake, Alberta, Canada.
**Table S2:** The individual monoterpenes (ng mg^−1^ fresh weight) in 30 families of *Picea glauca* foliage from Calling Lake, Alberta.
**Table S3:** The individual monoterpenes (ng mg^−1^ fresh weight) in 30 families/genotypes of *Picea glauca* foliage from Carson Lake, Alberta.
**Table S4:** The individual sesquiterpenes (ng mg^−1^ fresh weight) in 30 families of *Picea glauca* foliage from Calling Lake, Alberta.
**Table S5:** The individual sesquiterpenes (ng mg^−1^ fresh weight) in 30 families of *Picea glauca* foliage from Carson Lake, Alberta.
**Table S6.** Secondary metabolite profiles were identified in the mycelium of ten endophytic fungi of *Picea glauca* foliage.
**Table S7.** Concentrations of individual mycelium metabolite (ng mg−1 DE dry weight) in 10 endophytic fungi of *Picea glauca* foliage.
**Table S8.** Endophytic fungal volatile organic compounds were identified from ten endophytic fungi of *Picea glauca* foliage.
**Table S9.** Concentrations of individual volatile organic compounds (ng mg−1 dry weight) in 10 endophytic fungi of *Picea glauca* foliage. ND refers to not detected.
**Table S10.** The individual monoterpenes (ng mg^−1^) in 30 inoculated and control families/genotypes of *Picea glauca* seedling foliage.
**Table S11:** The individual sesquiterpenes (ng mg^‐1^) in 30 inoculated and control families/genotypes of *Picea glauca* seedling foliage.

## Data Availability

The data that support the findings of this study are available from the corresponding author upon reasonable request.
