# Supplementary material for: Alliance Between Conifer Trees and Endophytic Fungi Against Insect Defoliators
Source: Plant Cell Environ. 2025 Apr 1;48(7):5236–49. doi: 10.1111/pce.15503 (PMC12131960; doi:10.1111/pce.15503)
Supplement: Supplementary file 1 — Figure S1. Proportions of fungal guilds identified in Picea glauca families from Calling Lake and Carson Lake, Alberta (Canada) based on the FUNGuild database (Nguyen et al. 2016). Figure S2. Mean (±SE) endophytic fungal abundance (number of reads) observed among 30 Picea glauca phenotypes in (a) Calling Lake and (b) Carson Lake. Bars with different letters are statistically different (Tukey HSD tests). P‐value indicates the results of one‐way ANOVA. Calling Lake (a): F29,210=2.95, P < 0.001, n=4/family; Carson Lake (b): F29,197=1.99, P < 0.001, n=4/family. Figure S3. A heatmap showing the monoterpenes profiles of 30 families of Picea glauca that were sampled from Calling Lake, Alberta (Canada). The lowest to highest concentrations (ng mg−1 Fresh Weight) were demonstrated by light to dark colours, respectively. Concentrations of individual compounds were compared across families. Figure S4. A heatmap showing the monoterpenes profiles of 30 families of Picea glauca that were sampled from Carson Lake, Alberta (Canada). The lowest to highest concentrations (ng mg−1 Fresh Weight) were demonstrated by light to dark colours, respectively. Concentrations of individual compounds were compared across families. Figure S5. A heatmap showing the sesquiterpenes profiles of 30 families of Picea glauca that were sampled from Calling Lake, Alberta (Canada). The lowest to highest concentrations (ng g−1 Fresh Weight) were demonstrated by light to dark colours, respectively. Concentrations of individual compounds were compared across families. Figure S6. A heatmap showing the sesquiterpenes profiles of 30 families of Picea glauca that were sampled from Carson Lake, Alberta (Canada). The lowest to highest concentrations (ng mg−1 Fresh Weight) were demonstrated by light to dark colours, respectively. Concentrations of individual compounds were compared across families. Figure S7. Mean concentrations (±SE) of total monoterpenes and sesquiterpenes from different Picea glauca phenotypes in [file PCE-48-5236-s001.docx]

**Supplementary Information**

**Alliance Between Forest Conifer Trees and Endophytic Fungi Against Insect Defoliators**

Aziz Ullah^1,2^, Ateeq Shah^1^, Shih-hsuan (Ethan) Chen^1,3^, Aftab Shah^1^, Jean C. Rodriguez-Ramos^1,4^, Rashaduz Zaman^1,5^, Nadir Erbilgin^1*^

^1^Department of Renewable Resources, University of Alberta, Edmonton, AB, Canada

^2^ Department of Agricultural, Food and Nutritional Sciences, University of Alberta, Edmonton, AB, Canada

^3^ Current Address: Department of Pharmacy, University of Alberta, Edmonton, AB, Canada

^4^ Current Address: A&P Inphatec, LLC, Palo Alto, California, United States

^5^ Department of Biological Sciences, University of Alberta, Edmonton, AB, Canada

***Corresponding Author**: Nadir Erbilgin, Phone: (780)-492-6722; E-mail: [erbilgin@ualberta.ca](mailto:erbilgin@ualberta.ca)

**Supplementary Methods**

***DNA extraction and Illumine sequencing***

PCR reactions were prepared according to Platinum™ SuperFi™ Green PCR Master Mix (Invitrogen, Carlsbad, CA, USA) specifications and using 1µL of DNA extract and ran using a MyCycler™ Thermal Cycler (Eppendorf AG 22331 Hamburg, GER). The first fITS7 PCR was performed with an initial denaturation at 95°C for 2 min, followed by 35 cycles of 95°C for 30 s, 58°C for 30 s, and 68°C for 1 min, with a final extension at 68°C for 7 min. We used Mag-Bind® Total Pure NGS to purify the PCR products of each reaction before conducting an indexing PCR. We utilized TotalPure NGS (Omega Bio-Tek, Norcross, GA, United States) and 2.5 μl of each reaction to enable multiplexed sequencing for indexing PCR. The Nextera XT Index Kit (Illumina) was combined with the same polymerase enzyme for this second PCR, which was conducted under the following conditions: 95°C for 3 min followed by eight cycles at 95°C, 55°C and 72°C for 30 s each, and 72°C for 5 min. Again, we purified PCR products in the previously described manner and utilized 5 ul of each sample pooled together for two separate sequencing runs, each containing 240 samples. We examined the size and concentration of the pooled second PCR result using an Agilent 2100 Bioanalyzer (Santa Clara, CA, USA). We divided them into two amplicon libraries, each with 240 samples, four positive and four negative controls. Positive controls included DNA from one fungal species, whereas negative controls did not contain any fungal DNA to identify potential contamination during the procedure.

We submitted the DNA to the Applied Genomics Core (TAGC) at the University of Alberta for sequencing on the Illumina MiSeq platform using 2 × 300 bp paired-end reads with v3 chemistry. Sequence data was deposited in the NCBI (BioProject ID: PRJNA952578).

***Extraction of foliar terpenes***

We extracted the fresh needle tissue twice in 0.5 ml hexane with an internal standard of 0.004% pentadecane. Samples were vortexed for 30 s, sonicated for 10 min, and centrifuged at 16,100 rcf at 0°C for 15 min. We combined the supernatant from each extraction and identified monoterpene compounds in a sub-sample of extracts with a Gas Chromatograph/ Mass Spectrometer (GC/MS, Agilent 7890A/5062C, Agilent Tech., Santa Clara, CA, USA) using authentic standards. To quantify monoterpene and sesquiterpenes in all the samples, we used a GC/Flame Ionization Detector (GC/FID, Agilent 7890B). The method used for GC/MS was as follows: 1 μl of sample extract was injected with a split injection (10:1) into the GC equipped with DB-5MS UI column (30 m x 0.25 mm ID x 0.25 μm film, product: 122-5532UI; Agilent Tech.) with helium carrier gas flow at 1.1 ml min^-1^, and a temperature of 40 °C for 1 min, increased to 55 °C by 30 °C min^-1^ and held for 0.5 min, increased to 122 °C by 8 °C min^-1^ and held for 2 min, increased to 200 °C by 10 °C min^-1^, and then to 260 °C by 20 °C min^-1^ and held for 1 min. For monoterpenes analysis we used 11 authentic standards (mainly monoterpenes and bornyl acetate (acetate ester of borneol)) to identify and quantify the concentrations of individual compounds: borneol (chemical purity: 99%), α-terpineol (90%) (Sigma-Aldrich), terpinolene (90%), α-pinene (98%), β-pinene (98%), limonene (99%), myrcene (90%), camphene (90%), (+/-)-camphor (95%) (Fluka, Sigma-Aldrich, Buchs, CHE), bornyl acetate (97%), (SAFC Supply Solutions, St. Louis, MO, USA), and β-phellandrene (99%) (TRC Toronto, Canada). Units for monoterpenes (ng mg^-1^) were based on fresh weight (FW). For sesquiterpene analysis, we used β-caryophyllene (80%), caryophyllene oxide (95%), and (+) aromadendrene (97%) (Sigma-Aldrich). Units for sesquiterpenes (ng mg^-1^) were based on FW.

***Sanger sequencing and bioinformatics of cultured fungal endophytes***

PCR was conducted in 25 uL reactions to amplify the internal transcribed spacer (ITS) region of the retrieved nuclear rDNA (ITS1 and ITS2) using 1.0 μL of DNA extract, 6.5 μL of autoclaved deionized water, 12.5 μL of PlatinumTM SuperFiTM Green PCR Master Mix (Invitrogen), 2.5 L of 10 M ITS1-F, and 2.5 L of 10 M ITS-4 (Gardes & Bruns, 1993). The following thermal cycling parameters were used: a preliminary denaturation at 95°C for 5 min, 40 cycles of denaturation (95°C for 90 s, annealing at 55°C for 1 min, extension at 72°C for 90 s), and final extension at 72°C for 10 min. Amplification was confirmed using 1.7% agarose gel electrophoresis. For subsequent investigation, only samples that generated distinct single bands were chosen. ExoSAP IT (New England Biolabs, Ipswich, MA, USA) was used to enzymatically clean and purify amplified products. Cycle sequencing was carried out in 10 μL reactions that contained either the forward primer ITS1-F or the reverse primer ITS-4 at a concentration of 0.5 uM, 1 uL of cleaned PCR product, 0.5 UL of Big Dye Terminator v3.1 Ready Reaction Mix, and 1.5 uL of 5x Sequencing Buffer (Applied Biosystems, Foster City, CA, USA). The following were the conditions for thermal cycling that were applied to cycle sequencing reactions: initial denaturation at 96 degrees Celsius for one min, followed by 35 cycles of denaturation at 96 °C for 30 s, annealing at 50 °C for 15 s, and extension at 60 °C for 2 min. Ethanol precipitation was used to clean the sequencing experiments, and then the reactions were conducted on an ABI 3730 DNA analyzer (Applied Biosystems, Foster City, CA, USA).

Sequences were modified manually in Geneious prime (Biomatters Ltd, Auckland, NZL) following Taylor & Houston's method (Taylor & Houston, 2011). Briefly, bases with phred scores < 20 were transformed to Ns, and sequence ends cut to 3% error probability. BioEdit 7.2.5 was used to remove sequences with > 2% Ns. CAP3 was used to classify sequences into OTUs at 97% sequence identity with the following non-default settings: maximum overhang percentage length = 60; match score factor = 5; overlap percentage identity threshold = 96; clipping range = 6. The nucleotide BLAST program was used to compare consensus sequences from each OTU to those stored in the GenBank database. The BLAST results from the query dataset were further investigated for anomalies, and sequences that were probable assembly chimeras were removed from consideration. We used ≥ 97% sequence similarity to make taxonomic matches as this was shown to be a good estimate for fungi (Taylor *et al.*, 2014) (Table S1).

***Extraction of fungal metabolites from cultured fungal endophytes***

Samples were vortexed for 30 s, sonicated for 10 min, and centrifuged at 16,100 rcf at 0°C for 15 min. Using authentic standards, we combined the supernatant from each extraction and identified metabolites in a sub-sample of extracts with a GC/MS. The GC connected to a mass spectrometer equipped with a DB-5MS UI column was used to analyze the extracts (GC-MS; GC: 7890A, MS: 5062C, 30 m x 0.25 mm ID x 0.25 m film, product: 122-5532UI; Agilent Tech).

Helium was used as a carrier gas flowing at 1 mL min-1 with a temperature program beginning at 45°C to 50°C (held for 2 min), followed by an increase of 3°C min^-1^ to 70°C, then 5°C min^-1^ to 130°C, after that 12°C min^-1^ to 170°C, and finally the column temperature was brought to 300°C (held 2 min) at a rate of 30°C min^-1^. A 1 μl sample injection volume was used, the injector temperature was 250°C, and samples were run in splitless mode. We used seven standards to determine the concentrations of individual fungal metabolites, including ergosterol (chemical purity: 95%), farnesol (99%), β-caryophyllene (80%), dodecanoic acid (99%), methyl linoleate (99%), and methyl oleate (99%) (Sigma-Aldrich). Units were ng mg^-1^ dry weight (DW).

***Chemical analysis of fungal VOCs***

To identify fungal VOCs, the extracts were examined by GC-MS using a DB-5MS UI column (GC-MS; GC: 7890A, MS: 5062C, 30 m x 0.25 mm ID x 0.25 μm film, product: 122-5532UI; Agilent Tech). Helium was employed as a carrier gas flowing at 1 mL min^−1^ with a temperature program starting at 40°C (held for 1 min), 3 °C min^−1^ to 70 °C, 8 °C min^−1^ to 122 °C (held for 2 min), 10°C min^−1^ to 200 °C and 20°C min^−1^ to 325 °C and (held for 4 min). Samples were run splitless at 250 °C with a 1 ul injection volume. The control treatment peaks in the chromatograms of the fungal isolates were removed to identify the peaks that are particular to the medium. The following standards were used to verify and quantify library matches for all of the observed fungal volatiles using the NIST/EPA/NIH Mass Spectral library version 2.0f: α-pinene (chemical purity: 98%), β-pinene (98%), p-cymene (99%), camphene (90%), 3-carene (95%), iso-butanol (>99%), β-caryophyllene (80%), 3-methyl−1-butanol, dodecanoic acid (99%), (>99%) (Fluka, Sigma-Aldrich, Buchs, CHE), and β-phellandrene (99%) (Toronto Res. Chem.).

***Collection, extraction and analyses of fungal VOCs***

Two small Petri dishes (60 mm × 15 mm; Fisher Sci., Toronto, ON, Canada) with the same isolates were placed inside a volatile collecting chamber, a 473 mL glass jar with Teflon tape on its threads and a metal cover. Each collection was 10 times per isolate. We attached the jar with a vacuum pump (Cole-Parmer Canada Inc., Montreal, QC, CAN) and a flowmeter to maintain a constant airflow of 450 mL min^−1^ through the chamber lines. To filter and clean the air before it entered the collecting chamber, the intake channel was connected to a piece of Teflon tubing that was 30.5 cm long and filled halfway down with activated carbon (800 mg; 6–14 mesh; kept in place with glass wool) (air scrubber). The volatiles in the headspace were then collected for 12 h in a 7.5 cm plastic tube containing activated carbon (150 mg; 6−1 4 mesh, Fisher Sci.) and secured with glass wool at both ends. After the collecting time, pumps were turned off, trap tubes were removed and wrapped in labelled aluminum foil, and kept at −40°C until chemical extraction.

To extract the volatiles, 1 mL of dichloromethane containing tridecane as an internal standard (0.002%) was added to a microtube containing the activated carbon. Before collecting the extract and transferring it to a 2 ml glass GC vial, this combination was vortexed for 30 sec, sonicated for 10 min, and then centrifuged at an acceleration of 18,213 g for 30 min.

***Seedling fertilization and dormancy protocol***

The seeds were sown into 700 ml pots containing Sunshine Mix #4 (Sungro, Vilna, AB, CAN). The seedlings were grown in a growth chamber at the University of Alberta, and subjected to a 16/8 h photoperiod with maximum day and night temperatures of 24°C and 18°C, respectively. Seedlings were irrigated twice weekly. Beginning five weeks post-germination, seedlings were fertilized with 300 ppm phosphorus using a 10-52-10 (N:P:K) fertilizer mix and 18 ppm iron chelate every four weeks. Fertilizer was applied to avoid phosphorus and iron deficiencies that could be identified by the reddening and yellowing of seedling needles, respectively. Seedling dormancy protocol adapted from Kanekar *et al.* (2018). Seedlings were gradually acclimated to cold storage for two weeks; first stored at 15℃ during the day and 10℃ at night for the first week and then 6℃ for the second week. After seedlings were acclimated to the cold, they were stored at 4℃ for five weeks, and then the temperature in cold storage was gradually increased to 3℃ every day for a week until the temperature in the growth chamber reached 23℃. While dormancy was being simulated in the growth chamber, seedlings were watered weekly and kept under a 12h:12h (light:dark) light regime. No fertilization application took place during dormancy.

**Supplementary results**

***Foliage fungal community composition***

We sequenced 18.3 million DNA reads from two Illumina MiSeq runs, averaging 38,936 reads per sample. Following ‘DADA2’ quality control and filtering, 13.1 million DNA reads (12,681 ASVs) remained for subsequent analysis, representing 1,198 taxa. To ensure comparability, we subsampled 4,580 sequences per sample based on species rarefaction curves. Carson Lake had a higher proportion of endophytes than Calling Lake among 11 different fungal guilds identified (Fig. S1). Since we focused on endophytic fungi, we did not include other fungal guilds in the subsequent data analysis.

Carson Lake had a higher proportion of endophytes than Calling Lake among 11 different fungal guilds identified (Fig. S1). Furthermore, Carson Lake showed 45 endophytic fungal genera, while Calling Lake had 33 (Fig. 1). The five most abundant genera, *Cladosporium*, *Tryblidiopsis*, *Venturia*, *Lophodermium*, and *Lirula*, were consistent but varied in abundance between sites. *Tryblidiopsis*, *Venturia*, and *Lophedermium* were the most abundant in Carson Lake, whereas *Cladosporium* and *Lirula* were more prevalent in Calling Lake. Carson Lake also had a higher abundance (#reads) (815±49) than Calling Lake (606±31) (df=469, t=-3.51, *P*<0.001). Endophytic fungal abundance also varied among spruce families (Fig. S2).

Endophytic fungal abundance differed among spruce families (Fig. S2). In Calling Lake, 12 families had a mean read abundance of 500 or lower, while others ranged from 500 to 1,400. Families 1976 and 1951 showed the highest mean abundance, while families 178, 180, 195, 201, 203, 1982, and 1987 had the lowest abundance (Fig. S2a). Carson Lake showed similar patterns, with six families having a lower abundance of 500 or less and others ranging from 500 to 1,552 (Fig. S2b). Families 1580, 156, 180, 188, 190, 1976, and 1978 had the highest abundance, whereas families 143, 157, 158, 170, 176, 178, 1924, 1952, and 2106 showed the lowest fungal abundance.

***Terpene composition and variations across sites and spruce families***

Calling Lake had higher total monoterpene concentrations (2481 ± 125 ng mg^-1^) than Carson Lake (1448 ± 81 ng mg^-1^) (df=469, t=6.841, *P*<0.001). In contrast, Carson Lake (1335 ± 67 ng mg^-1^) had a higher total sesquiterpene concentration than Calling Lake (1784 ± 95 ng mg^-1^) (df=469, t=-3.867, *P*<0.001). The total concentrations of monoterpenes and sesquiterpenes varied among different spruce families at each site (Fig. S3-S7, Suppl Results).

The total concentrations of monoterpenes and sesquiterpenes varied among different spruce families at each site (Fig. S3-S7). In Calling Lake, three families showed a mean range of monoterpene concentrations ranging from 799 to 1,058 ng mg^-1^ and sesquiterpenes from 284 to 477 ng mg^-1^ (Fig. S3, S5, Fig. S7a). The remaining families had mean ranges from 1,058 to 4,803 ng mg^-1^ for monoterpenes and from 477 to 2,846 ng mg^-1^ for sesquiterpenes. In Carson Lake, the mean concentrations for 11 families ranged from 420 to 1,010 ng mg^-1^ for monoterpenes and from 117 to 721 ng mg^-1^ for sesquiterpenes, while other families had concentrations ranging from 1,010 to 3,276 ng mg^-1^ for monoterpenes and from 117 to 4,103 ng mg^-1^ for sesquiterpenes (Fig. S4, S6, Fig. S7b). Linear regression analyses revealed a positive correlation between monoterpenes and sesquiterpenes in both Calling Lake (*P*<0.001, R^2^=0.2; Fig. S8a) and Carson Lake (*P*<0.001, R^2^=0.85; Fig. S8b).

References

**Gardes M, Bruns TD**. **1993**. ITS primers with enhanced specificity for basidiomycetes‐application to the identification of mycorrhizae and rusts. *Molecular Ecology* **2**: 113–118.

**Kanekar SS, Cale JA, Erbilgin N**. **2018**. Ectomycorrhizal fungal species differentially affect the induced defensive chemistry of lodgepole pine. *Oecologia* **188**: 395–404.

**Taylor DL, Hollingsworth TN, McFarland JW, Lennon NJ, Nusbaum C, Ruess RW**. **2014**. A first comprehensive census of fungi in soil reveals both hyperdiversity and fine‐scale niche partitioning. *Ecological Monographs* **84**: 3–20.

**Taylor DL, Houston S**. **2011**. A bioinformatics pipeline for sequence-based analyses of fungal biodiversity. *Fungal Genomics: Methods and Protocols*: 141–155.


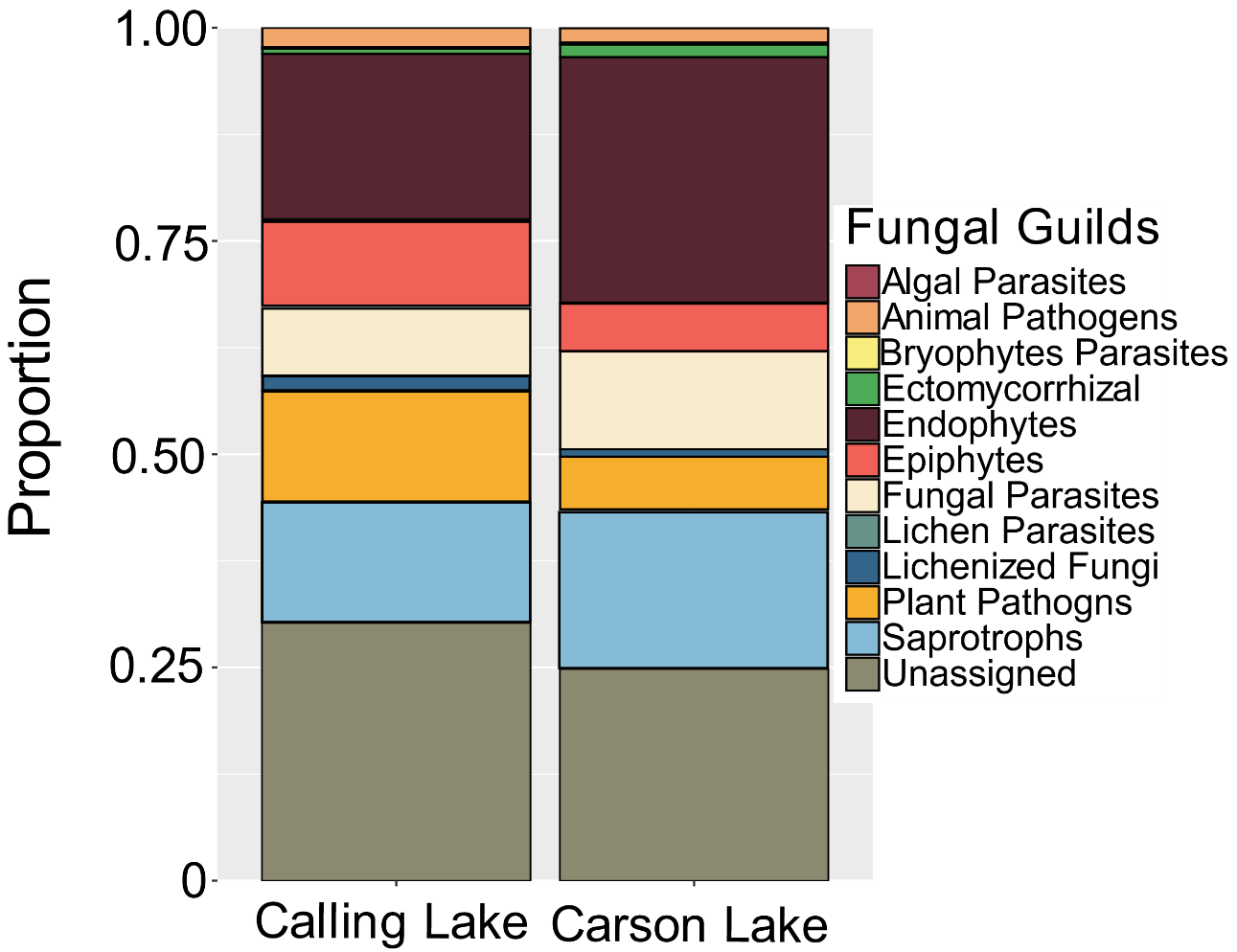


**Figure S1.** Proportions of fungal guilds identified in *Picea glauca* families from Calling Lake and Carson Lake, Alberta (Canada) based on the FUNGuild database (Nguyen *et al.,* 2016).

**
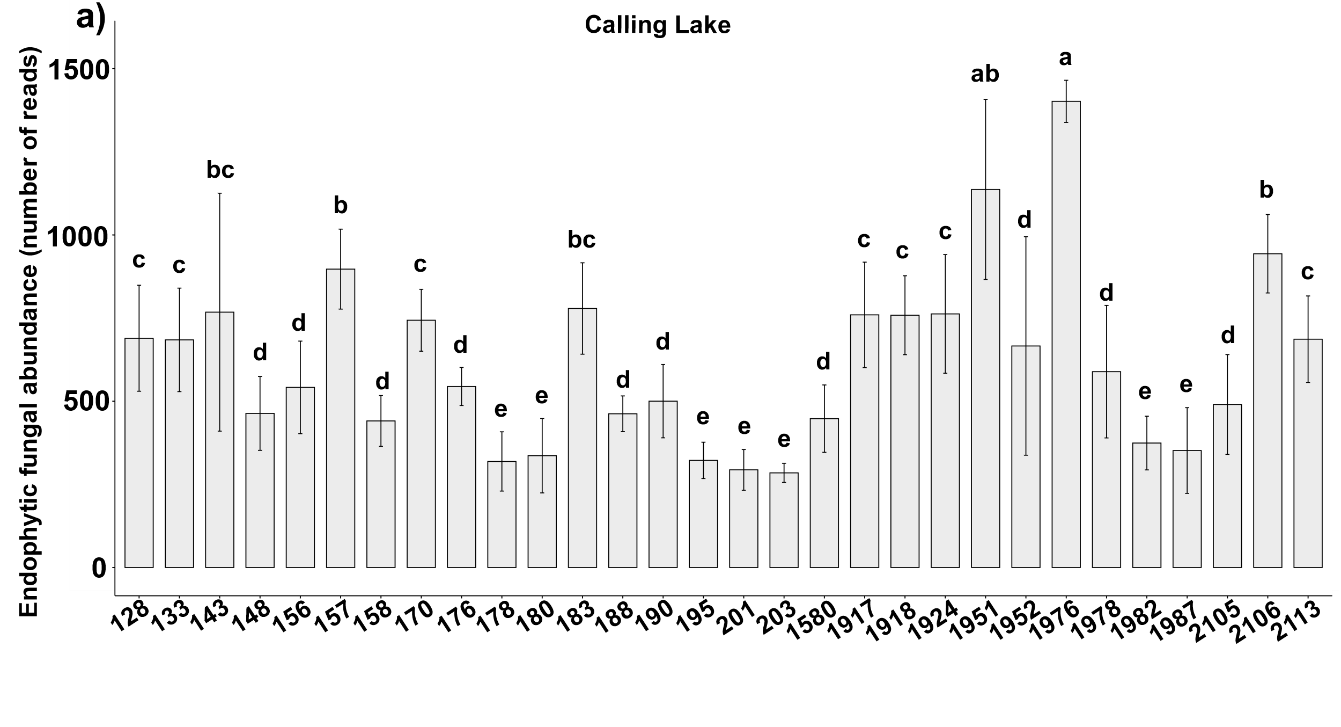
**


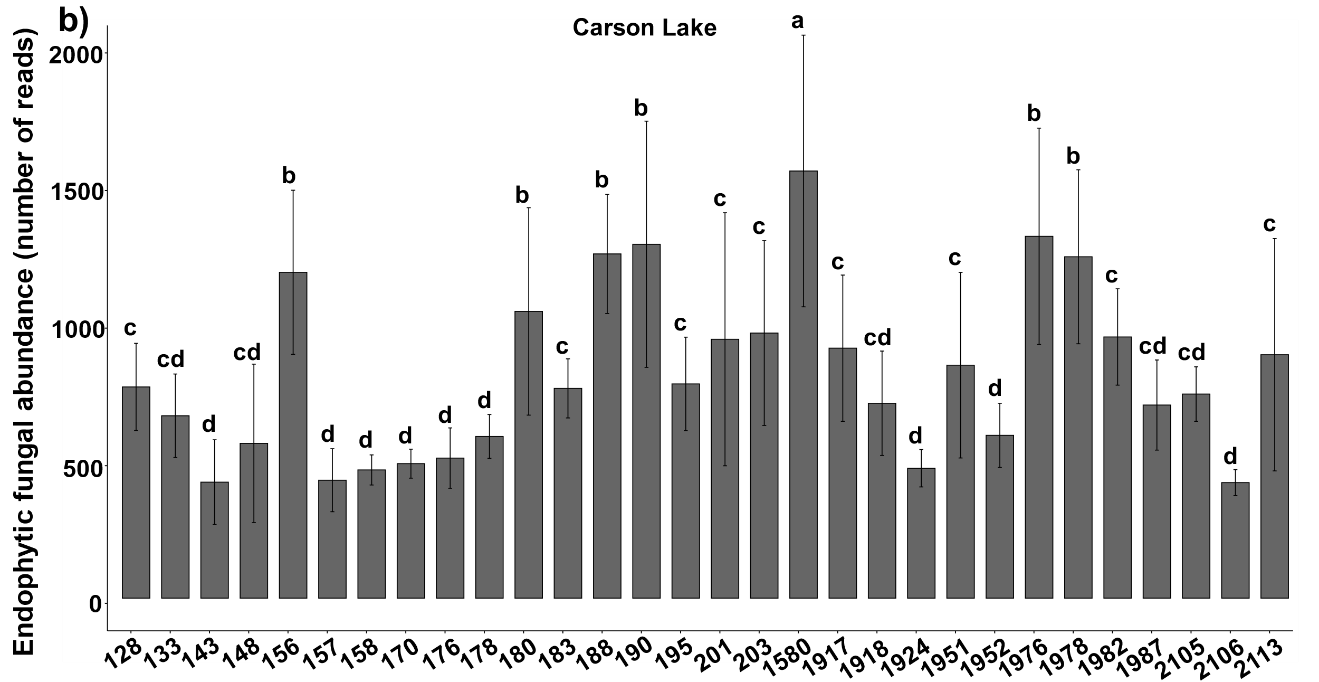


**Figure S2.** Mean (±SE) endophytic fungal abundance (number of reads) observed among 30 *Picea glauca* phenotypes in **(a)** Calling Lake and **(b)** Carson Lake. Bars with different letters are statistically different (Tukey HSD tests). *P*-value indicates the results of one-way ANOVA. Calling Lake (a): F_29,210_=2.95, *P* <0.001, n=4/family; Carson Lake (b): F_29,197_=1.99, *P* <0.001, n=4/family.


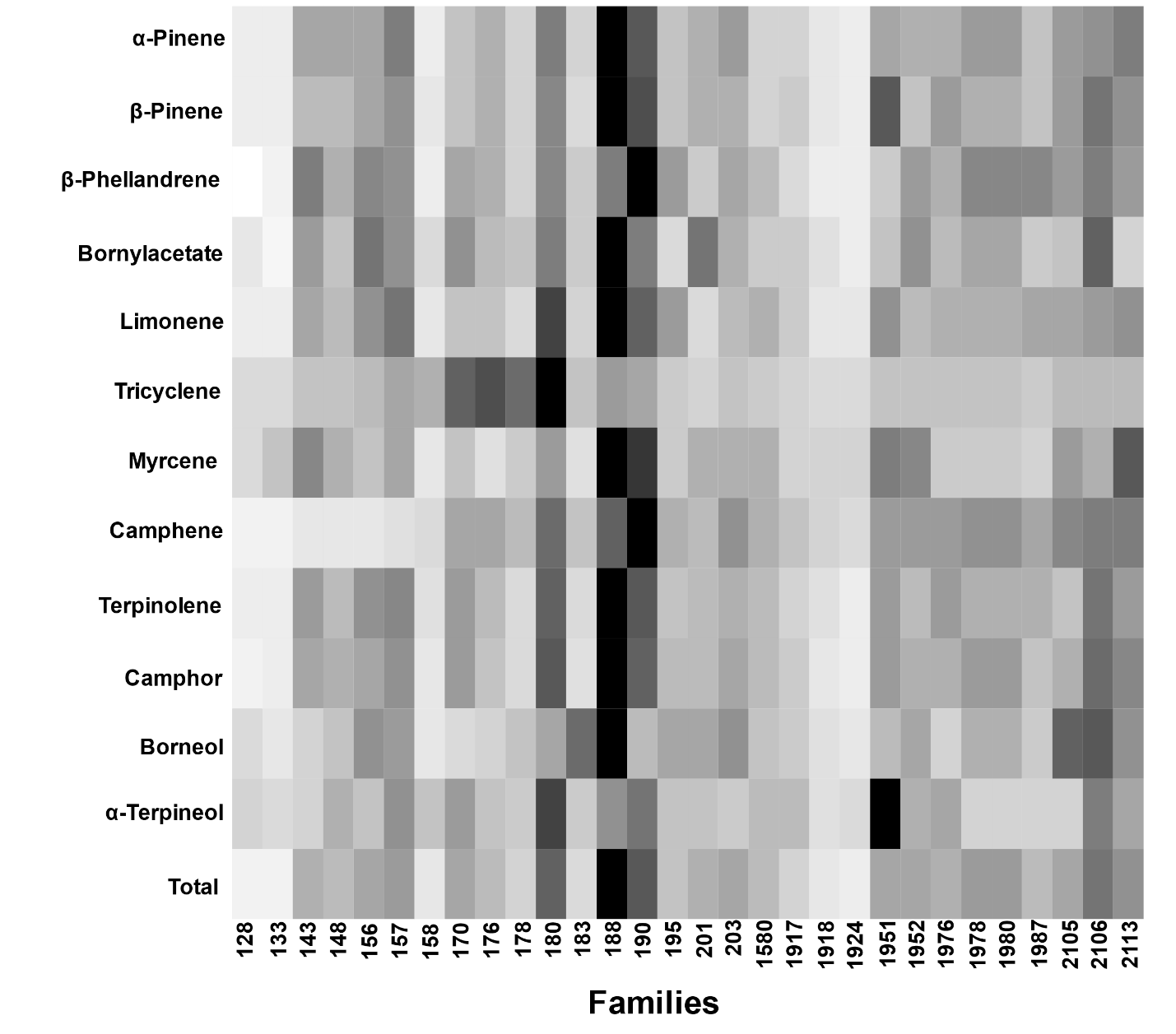


**Figure S3.** A heatmap showing the monoterpenes profiles of 30 families of *Picea glauca* that were sampled from Calling Lake, Alberta (Canada). The lowest to highest concentrations (ng mg^-1^ Fresh Weight) were demonstrated by light to dark colours, respectively. Concentrations of individual compounds were compared across families.


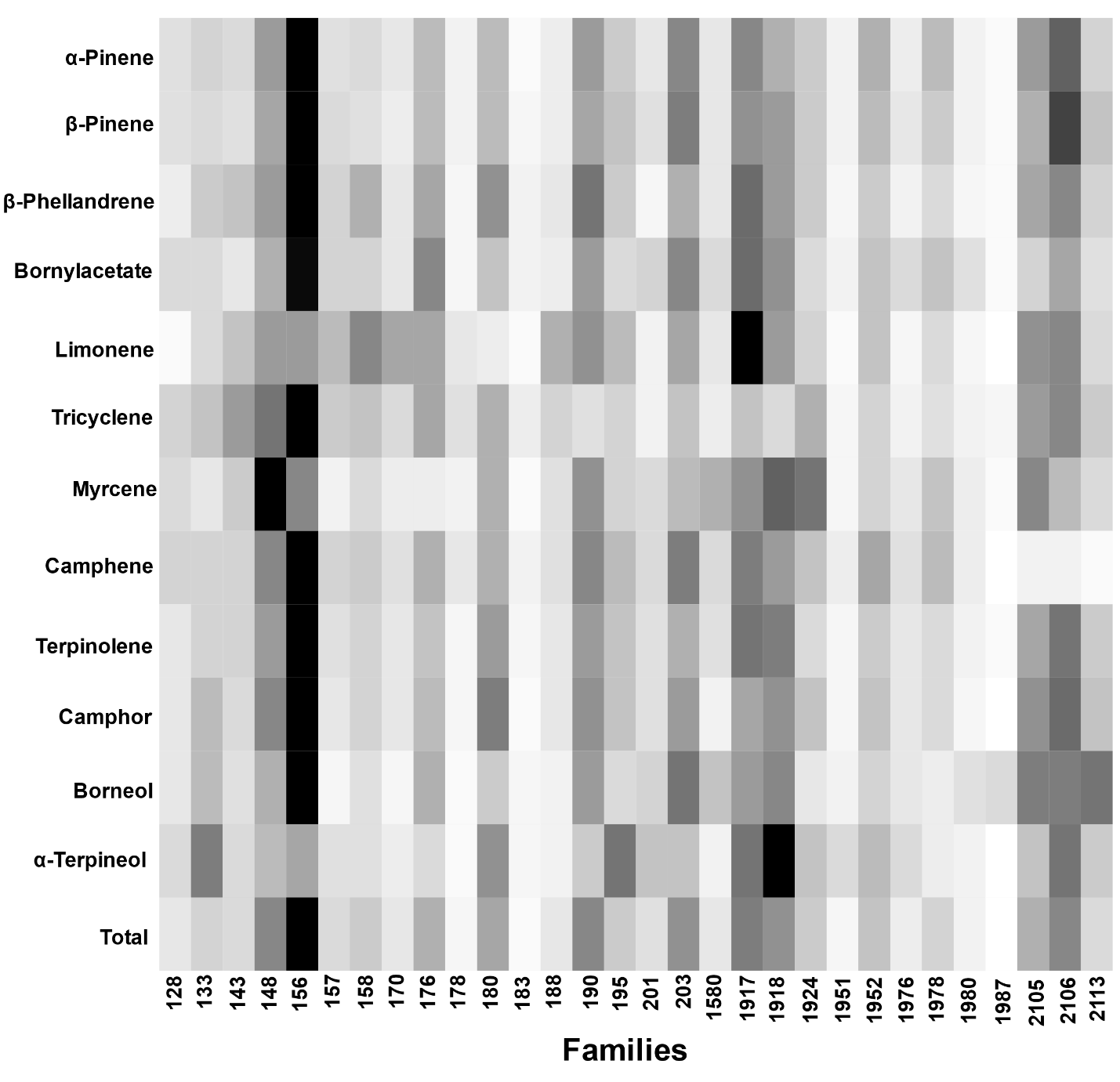


**Figure S4.** A heatmap showing the monoterpenes profiles of 30 families of *Picea glauca* that were sampled from Carson Lake, Alberta (Canada). The lowest to highest concentrations (ng mg^-1^ Fresh Weight) were demonstrated by light to dark colours, respectively. Concentrations of individual compounds were compared across families.


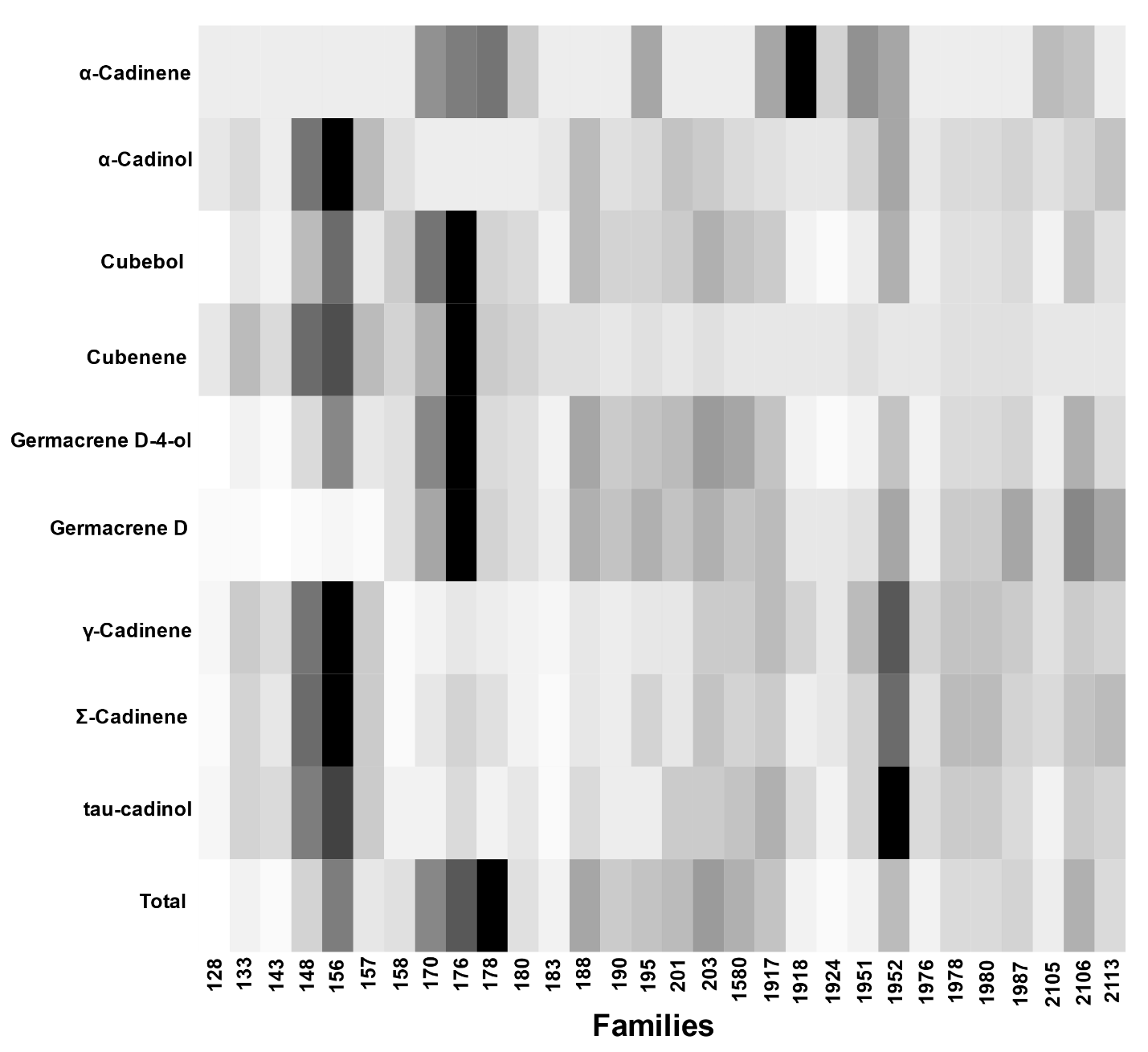


**Figure S5.** A heatmap showing the sesquiterpenes profiles of 30 families of *Picea glauca* that were sampled from Calling Lake, Alberta (Canada). The lowest to highest concentrations (ng g^-1^ Fresh Weight) were demonstrated by light to dark colours, respectively. Concentrations of individual compounds were compared across families.


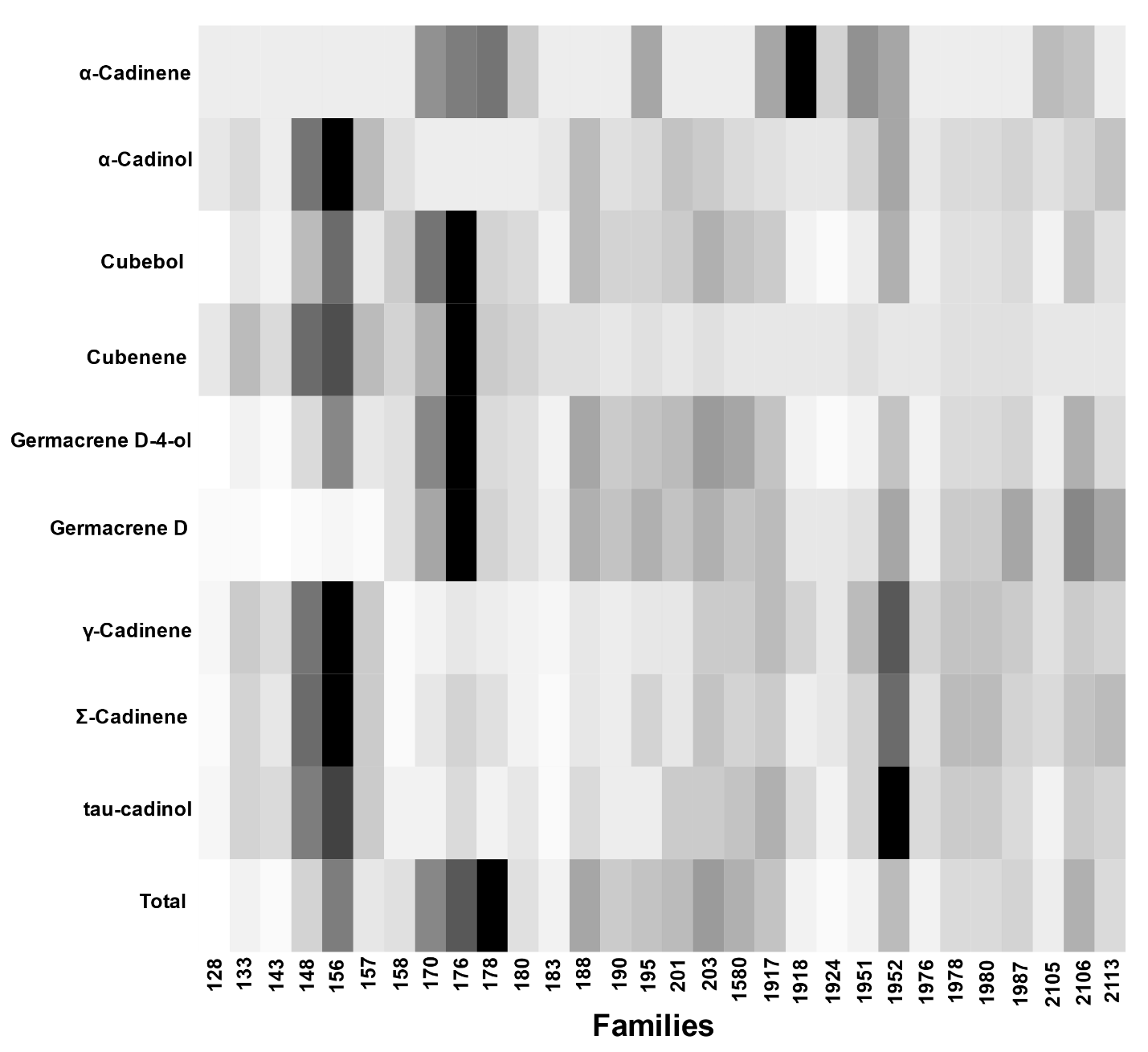


**Figure S6.** A heatmap showing the sesquiterpenes profiles of 30 families of *Picea glauca* that were sampled from Carson Lake, Alberta (Canada). The lowest to highest concentrations (ng mg^-1^ Fresh Weight) were demonstrated by light to dark colours, respectively. Concentrations of individual compounds were compared across families.


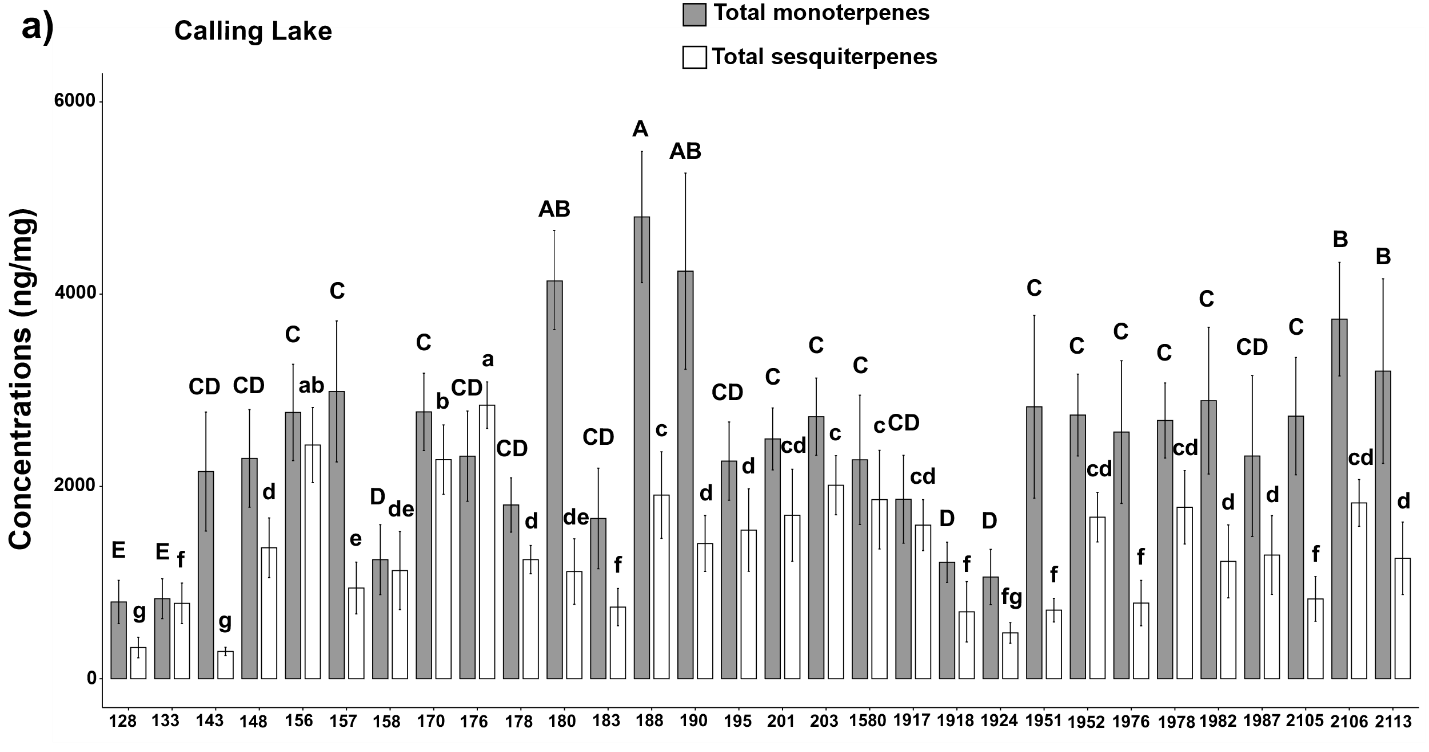


**
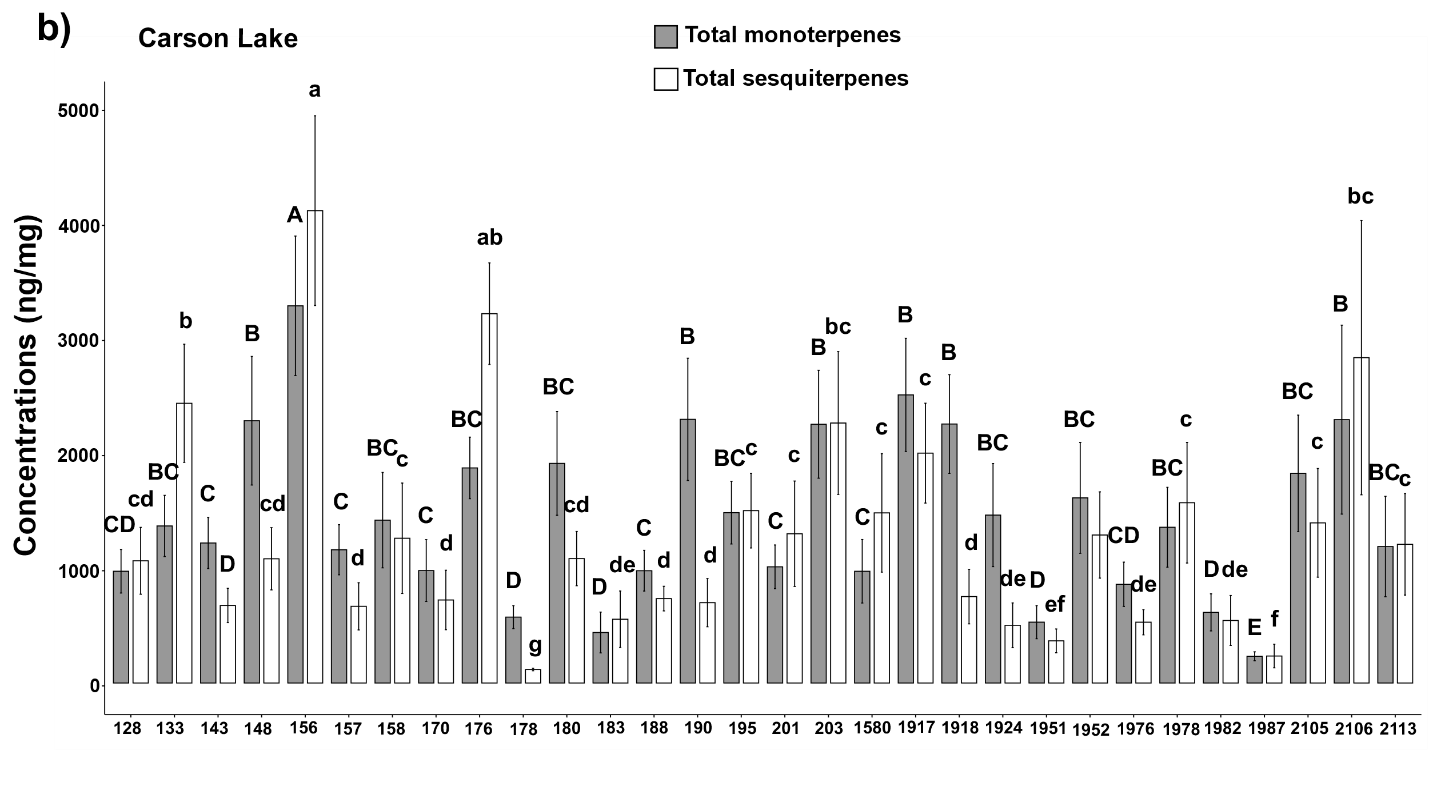
**

**Figure S7.** Mean concentrations (±SE) of total monoterpenes and sesquiterpenes from different *Picea glauca* phenotypes in **(a)** Calling Lake and **(b)** Carson Lake. Bars with different capital letters representing total monoterpenes and small letters representing total sesquiterpenes are statistically different (Tukey HSD tests). *P*-values indicate the results of one-way ANOVA. Calling Lake: Monoterpenes: F_29,210_=2.3, *P*<0.001; Sesquiterpenes: F_29,210_=3.94, *P*<0.001. Carson Lake: Monoterpenes: F_29,210_=3.99, *P*<0.001; Sesquiterpenes: F_29,210_=4.63, *P*<0.001.

**
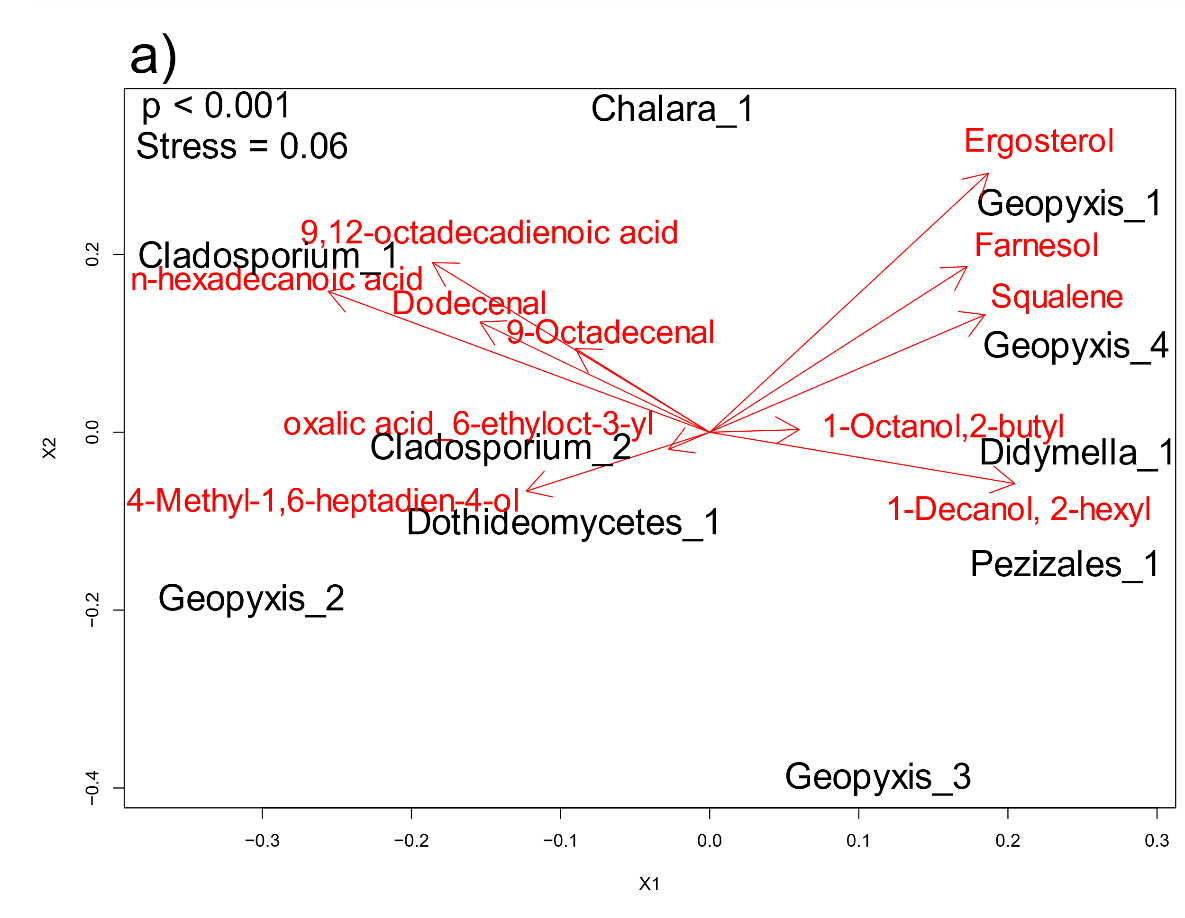
**
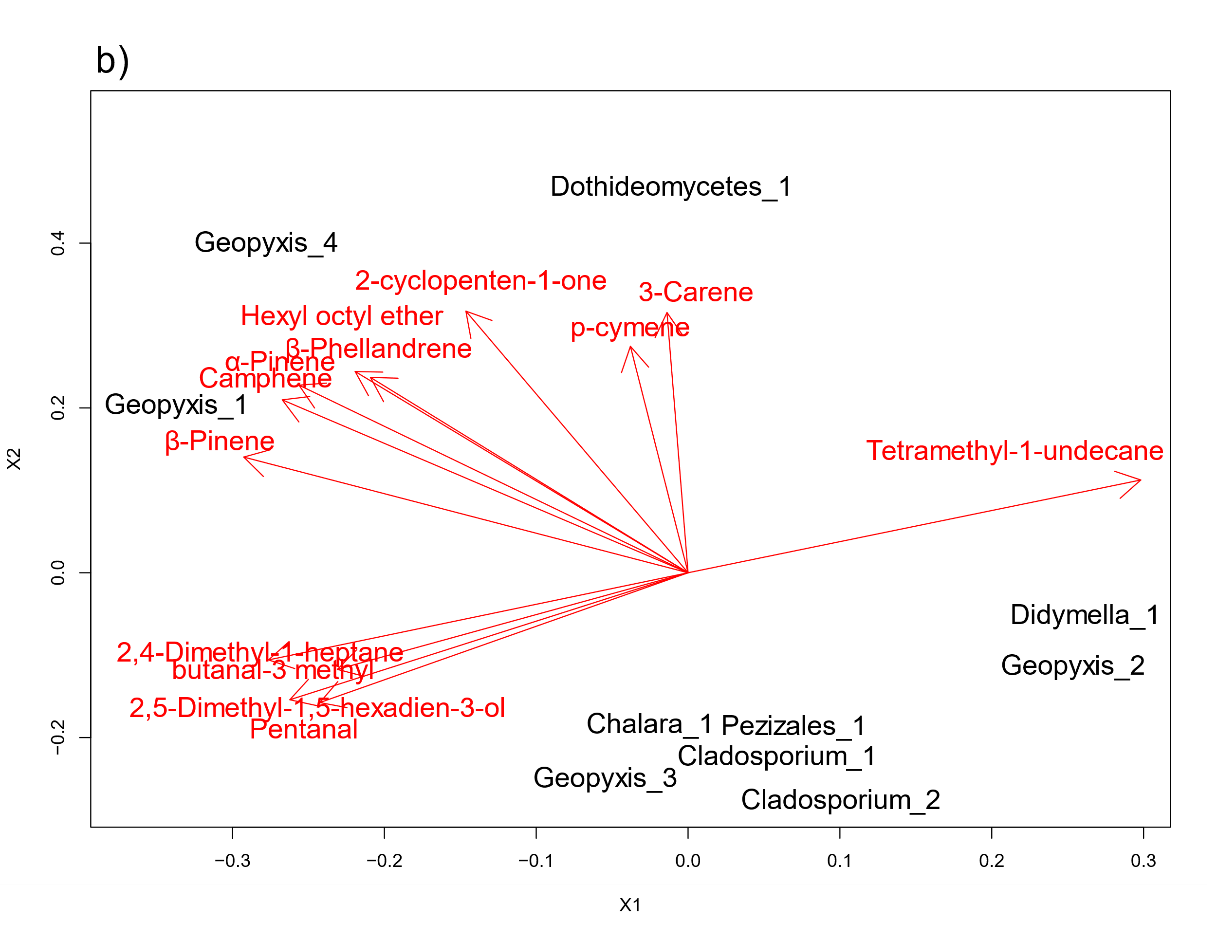


**Figure S8 (a)** An NMDS showing the distribution of fungal metabolites (red vectors) from 10 endophytic fungi (black text). **(b)** An NMDS plots the distribution of individual endophytic fungal volatile organic compounds abundance (red vectors) in different endophytic fungi (black text).


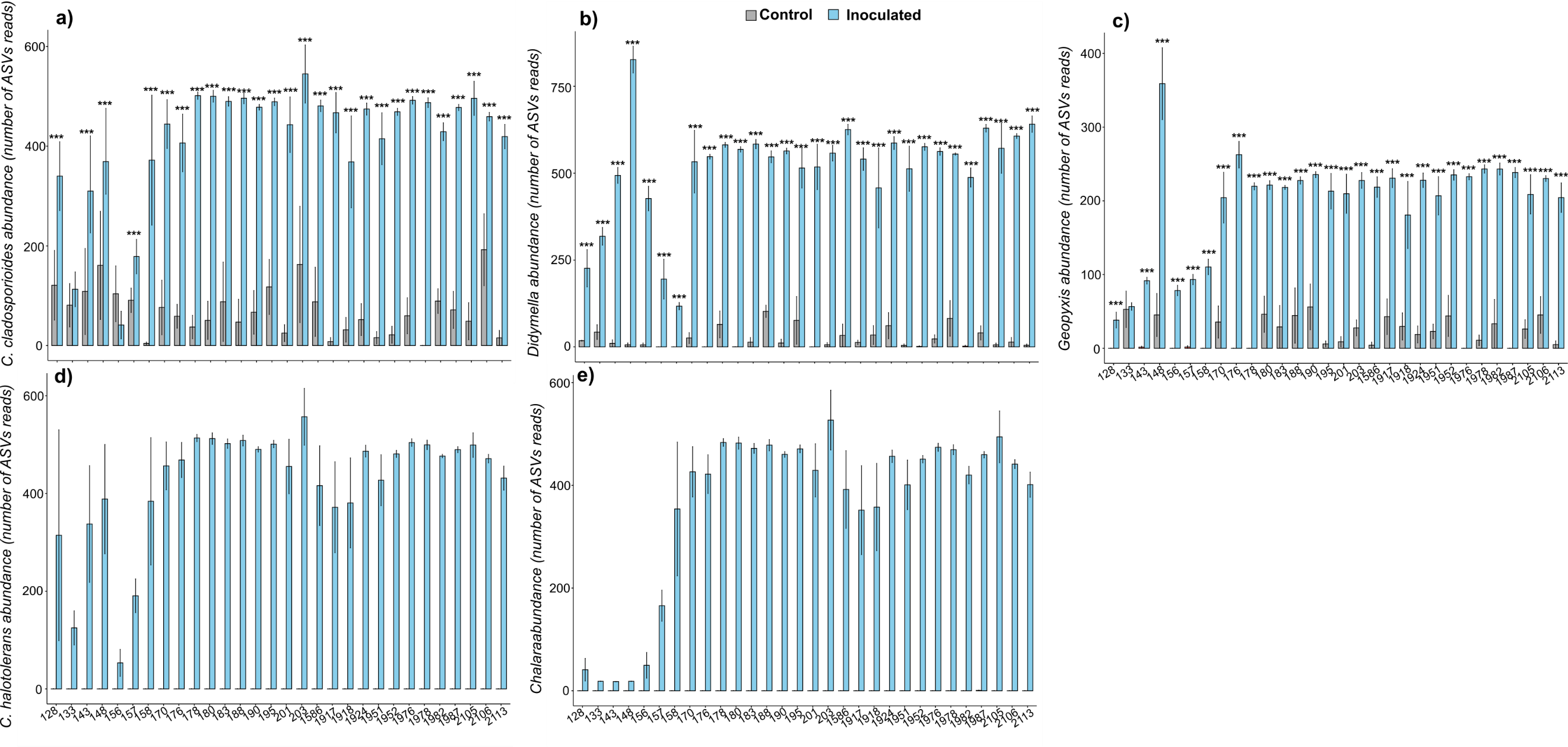


**Figure S9.** Mean (±SE) endophytic fungal read abundance of **(a)** *Cladosporium cladosporioides*, **(b)** *Didymella*, **(c)** *Geopyxis*, **(d)** *Cladosporium halotolerans*, and **(e)** *Chalara* among seedlings of 30 *Picea glauca* families. The two-sample t-test was conducted for individual comparisons of control and inoculated families. Asterisks show significant differences from the control seedlings at *p* < 0.01.


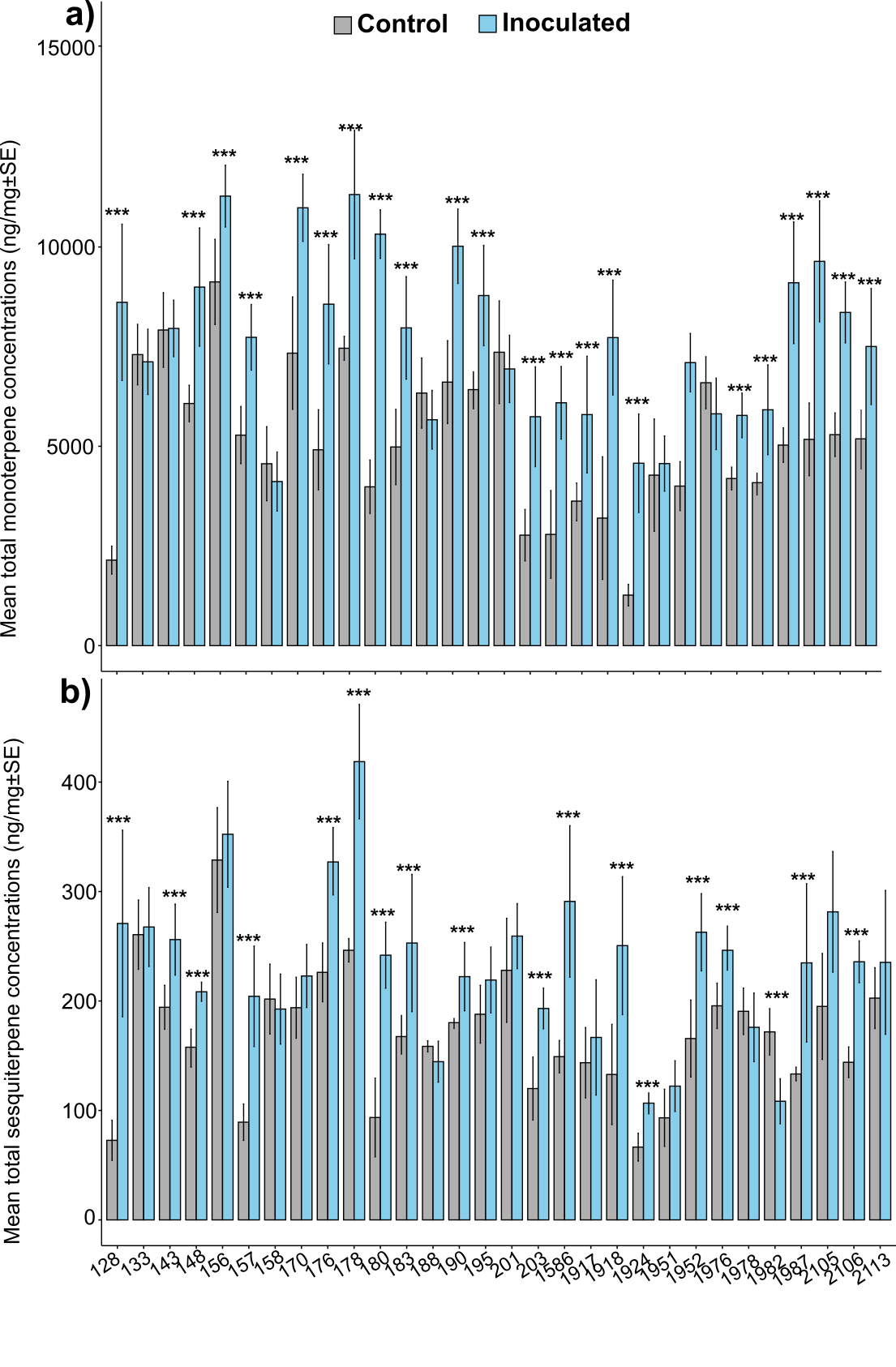


**Figure S10.** Mean concentrations (±SE) of **(a)** total monoterpenes and **(b)** total sesquiterpenes among families of *Picea glauca* seedlings. The two-sample t-test was conducted for individual comparisons of control and inoculated seedling families. Asterisks show significant differences from the control seedlings at *p* < 0.01.

**Table S1.** Operational taxonomic units (OTUs) of selected endophytic fungi found on *Picea glauca* foliage collected in Calling Lake and Carson Lake, Alberta, Canada.

| OTU | Best GenBank match | Best GenBank match  (Accession Number) | % Identity | Query coverage (%) |
| --- | --- | --- | --- | --- |
| *Chalara*_1 | *Chalara sp.* TMS-2011 voucher MS3p_50-44 | HQ630988 | 100.0 | 100.0 |
| *Cladosporium*_1 | *Cladosporium cladosporioides* | KU182497 | 100.0 | 100.0 |
| *Cladosporium*_2 | *Cladosporium halotolerans* | NR_119605 | 100.0 | 100.0 |
| *Didymella*_1 | *Didymella sp.* strain ICMP 12081 | MZ098689 | 98.6 | 99.0 |
| *Dothideomycetes*_1 | *Dothideomycetes sp.* 11143 | GQ153116 | 96.9 | 99.2 |
| *Geopyxis*_1 | *Geopyxis carbonaria* strain RAS132 | MG663262 | 100.0 | 100.0 |
| *Geopyxis*_2 | *Geopyxis carbonaria* voucher PRM149720 | KU932495 | 98.2 | 100.0 |
| *Geopyxis*_3 | *Geopyxis carbonaria* voucher DED 7357 (SFSU) | KU932486 | 99.1 | 99.1 |
| *Geopyxis*_4 | *Geopyxis carbonaria* voucher K (M)181130 | KU932489 | 97.0 | 98.0 |
| *Pezizales*_1 | *Pezizales sp.* T4N26c(A) | AY465510 | 97.3 | 98.5 |

| **Table S2:** The individual monoterpenes (ng mg^-1^ fresh weight) in 30 families of *Picea glauca* foliage from Calling Lake, Alberta. | | | | | | | | | | | | | |
| --- | --- | --- | --- | --- | --- | --- | --- | --- | --- | --- | --- | --- | --- |
| **Families** | **α-Pinene** | **β-Pinene** | **Β-Phellandrene** | **Bornyl**  **acetate** | **Limonene** | **Tricyclene** | **Myrcene** | **Camphene** | **Terpinolene** | **Camphor** | **Borneol** | **α -Terpineol** | **Total** |
| **128** | 87.7 | 15.9 | 17.9 | 195 | 67.3 | 5.3 | 48.9 | 24.6 | 10.3 | 312 | 9.4 | 4.9 | 799 |
| **133** | 95.2 | 15.6 | 47 | 78.3 | 86.6 | 4.9 | 84.1 | 25.7 | 9.4 | 377 | 5.08 | 4 | 833 |
| **143** | 230.6 | 37.1 | 161.9 | 522.4 | 286 | 11.4 | 144.5 | 64.4 | 35.5 | 1127.4 | 12.5 | 5.8 | 2639.7 |
| **148** | 241.6 | 39.1 | 123.2 | 354.8 | 252.2 | 12 | 108.6 | 66.7 | 26.9 | 1039.5 | 17.1 | 10.2 | 2292 |
| **156** | 235.4 | 44.9 | 153.3 | 663.5 | 357.6 | 12.9 | 82.4 | 69.1 | 37.9 | 1076 | 28.5 | 8.3 | 2770 |
| **157** | 296.8 | 51.48 | 145.4 | 535.9 | 421.8 | 17.96 | 113.9 | 109.3 | 40.12 | 1216 | 26.03 | 13.61 | 2988 |
| **158** | 93.8 | 16.3 | 54.3 | 250 | 94.7 | 15.7 | 33 | 148 | 14.2 | 506 | 6.2 | 7.9 | 1239 |
| **170** | 187.5 | 35.4 | 134.1 | 553.9 | 231.2 | 32.3 | 83.2 | 312.8 | 35.5 | 1146 | 11.1 | 13.3 | 2776 |
| **176** | 218 | 41.2 | 125 | 407 | 225 | 36.1 | 34.9 | 326 | 28 | 853 | 12.7 | 8.4 | 2315 |
| **178** | 155.7 | 27.1 | 87.3 | 356.7 | 158.3 | 29.3 | 71.7 | 251 | 18.1 | 628.9 | 17.2 | 6.3 | 1808 |
| **180** | 303 | 54.9 | 157 | 604 | 554 | 51.5 | 130 | 485 | 51.5 | 1697 | 24.8 | 24.4 | 4138 |
| **183** | 148.9 | 25.9 | 99.9 | 331.1 | 170.2 | 12.1 | 44 | 231.5 | 18.9 | 540.6 | 37.7 | 6.5 | 1667 |
| **188** | 414 | 75 | 168 | 860 | 505 | 19.9 | 249 | 512 | 63.7 | 1870 | 51.2 | 14.6 | 4804 |
| **190** | 367.2 | 74.1 | 182.4 | 622.8 | 478.7 | 16.9 | 234.9 | 548.7 | 53.8 | 1622 | 20.4 | 18 | 4239 |
| **195** | 191 | 34.3 | 138 | 257 | 311 | 9.3 | 69.5 | 293 | 26.8 | 900 | 25.1 | 7.9 | 2264 |
| **201** | 213.9 | 41.9 | 102.4 | 643.3 | 159.5 | 7.1 | 101.6 | 249.3 | 27.4 | 915.2 | 24.7 | 8.2 | 2494 |
| **203** | 247.6 | 41.8 | 133.1 | 431.5 | 255.5 | 11.8 | 109.8 | 366.2 | 29.8 | 1063 | 28.7 | 6.9 | 2726 |
| **1580** | 159.8 | 28.5 | 118.7 | 341 | 275.3 | 9.1 | 108.7 | 270.8 | 27 | 912.9 | 16.4 | 9.3 | 2278 |
| **1917** | 150 | 30.4 | 85.5 | 305.7 | 198.4 | 7.3 | 57.9 | 238.1 | 21 | 750 | 13.9 | 8.5 | 1867 |
| **1918** | 101.7 | 18.8 | 53.7 | 205.2 | 99.2 | 5 | 58.4 | 165.6 | 14.2 | 479.4 | 8.1 | 2.9 | 1212 |
| **1924** | 97.1 | 15.7 | 57.2 | 141.9 | 104.5 | 4.7 | 64.8 | 148 | 8.8 | 406.3 | 5.2 | 3.8 | 1058 |
| **1951** | 238.3 | 69.8 | 98.1 | 343.1 | 334.7 | 11 | 160.2 | 343.5 | 37.1 | 1140.9 | 20.4 | 31.5 | 2828.7 |
| **1952** | 214.2 | 35.3 | 136.8 | 533.6 | 252.2 | 10.2 | 151.2 | 336.9 | 27.9 | 1010 | 23.9 | 10.3 | 2743 |
| **1976** | 226.6 | 47.1 | 121.7 | 397.2 | 266.4 | 10.7 | 69.1 | 348.7 | 35.3 | 1019.1 | 12.3 | 11.9 | 2566.1 |
| **1978** | 245.9 | 40.9 | 157.8 | 485.8 | 283.9 | 11.7 | 77.1 | 381.3 | 30.1 | 1151 | 21.3 | 5.8 | 2892.5 |
| **1980** | 245.9 | 40.9 | 157.8 | 485.8 | 283.9 | 11.7 | 77.1 | 381.3 | 30.1 | 1151 | 21.3 | 5.8 | 2892.5 |
| **1987** | 188.3 | 32.8 | 158.9 | 341.8 | 286.8 | 9.2 | 60.9 | 304.6 | 30.7 | 883.1 | 14.9 | 5.1 | 2317.1 |
| **2105** | 257.5 | 49 | 140.5 | 365.4 | 292.5 | 14.2 | 127.9 | 389.7 | 26.7 | 1022 | 40 | 5.6 | 2732 |
| **2106** | 270.8 | 61.4 | 163.2 | 735.4 | 314.9 | 12.8 | 101.1 | 429.8 | 46.2 | 1545 | 43.6 | 16.4 | 3741 |
| **2113** | 298.3 | 51.6 | 141.4 | 285.9 | 337.3 | 13.7 | 206.6 | 441.8 | 37.3 | 1345 | 29.6 | 11.1 | 3200 |

| **Table S3:** The individual monoterpenes (ng mg^-1^ fresh weight) in 30 families/genotypes of *Picea glauca* foliage from Carson Lake, Alberta. | | | | | | | | | | | | | |
| --- | --- | --- | --- | --- | --- | --- | --- | --- | --- | --- | --- | --- | --- |
| **Families** | **α-Pinene** | **β-Pinene** | **Β-Phellandrene** | **Bornyl**  **acetate** | **Limonene** | **Tricyclene** | **Myrcene** | **Camphene** | **Terpinolene** | **Camphor** | **Borneol** | **α -Terpineol** | **Total** |
| **128** | 84.9 | 16.5 | 38.9 | 264.1 | 51.4 | 5.8 | 50.7 | 133.1 | 10 | 306 | 5 | 4 | 970.7 |
| **133** | 102.3 | 18.1 | 80.5 | 234.8 | 144.1 | 6.8 | 38.7 | 141.1 | 14.5 | 561.7 | 12.7 | 8.8 | 1364.3 |
| **143** | 91.4 | 16 | 86.6 | 196.1 | 183.6 | 9.5 | 68.1 | 140.4 | 14 | 400.4 | 6.6 | 3.9 | 1216 |
| **148** | 168.1 | 29.9 | 112.5 | 423.5 | 257.2 | 12 | 159.3 | 277.9 | 24.9 | 793.9 | 13 | 6 | 2279 |
| **156** | 270.8 | 50.9 | 173.3 | 940.2 | 243.3 | 19.5 | 122.4 | 427.9 | 41.5 | 956.6 | 23.6 | 6.7 | 3277 |
| **157** | 88.8 | 17.4 | 68.5 | 274.3 | 208 | 6 | 24.7 | 136.8 | 10.7 | 317.1 | 1.9 | 3.5 | 1157.6 |
| **158** | 97.2 | 16.6 | 98.6 | 268.8 | 281.4 | 6.9 | 49.3 | 156.9 | 14.7 | 414.7 | 6 | 3.6 | 1415 |
| **170** | 72 | 11.8 | 45.9 | 167.1 | 231.2 | 5 | 31.3 | 109.9 | 8.6 | 290.2 | 1.3 | 2.5 | 976.9 |
| **176** | 131.9 | 26 | 110.6 | 536.1 | 242.3 | 8.6 | 32.6 | 201.2 | 18.3 | 543.2 | 13.6 | 4 | 1869 |
| **178** | 58.1 | 9.8 | 21.9 | 85.2 | 106.1 | 4.4 | 22.1 | 93.4 | 3.9 | 165.9 | 1.1 | 0.7 | 572.4 |
| **180** | 130.4 | 26.2 | 120.7 | 340.2 | 95.8 | 8.1 | 89 | 205.7 | 24.6 | 849.6 | 9.6 | 7.9 | 1908 |
| **183** | 37.7 | 6.8 | 27.9 | 107.7 | 49.7 | 3.2 | 10.4 | 61.1 | 4.3 | 127 | 1.6 | 1.3 | 438.8 |
| **188** | 68.5 | 11.7 | 45.8 | 150 | 217 | 6 | 42.4 | 108 | 9 | 311 | 3.2 | 2 | 976 |
| **190** | 165.7 | 29 | 148.7 | 500.5 | 265.5 | 4.4 | 115.2 | 270.7 | 23.8 | 746.8 | 15.4 | 4.8 | 2291 |
| **195** | 118 | 24.7 | 80.5 | 241 | 210 | 5.8 | 56 | 186 | 17.2 | 522 | 7.7 | 9.5 | 1480 |
| **201** | 76 | 15 | 25.8 | 269 | 67.9 | 2.8 | 50.7 | 118 | 10.7 | 360 | 9 | 5.2 | 1010 |
| **203** | 188.4 | 37.4 | 103.1 | 556.9 | 229.5 | 6.8 | 84.8 | 279.3 | 20.6 | 715.1 | 20.5 | 5.5 | 2248 |
| **1580** | 78 | 13 | 48.1 | 264.8 | 104.9 | 3.1 | 86 | 124.3 | 10.5 | 224.5 | 11.4 | 2.1 | 970.7 |
| **1917** | 179 | 33 | 153.6 | 647.3 | 358.3 | 6.9 | 114.8 | 290.8 | 30.5 | 664.2 | 15.2 | 9.4 | 2503 |
| **1918** | 142.2 | 31.8 | 113.6 | 512.9 | 244 | 5.4 | 147.2 | 234.6 | 28.7 | 759 | 18.2 | 12.3 | 2250 |
| **1924** | 116.7 | 21.5 | 74.4 | 248.3 | 159.7 | 8.2 | 131.8 | 173.2 | 13.2 | 500.9 | 5.7 | 5.2 | 1458.8 |
| **1951** | 50.3 | 9.7 | 20.7 | 127.2 | 46.3 | 1.8 | 17.9 | 77.5 | 4.7 | 165.6 | 2.9 | 3.9 | 528.5 |
| **1952** | 144.4 | 24.8 | 74 | 355.7 | 181.5 | 5.6 | 61.6 | 220 | 15.6 | 509.6 | 9.3 | 6 | 1608 |
| **1976** | 69.4 | 14 | 29.3 | 244.1 | 59.1 | 2.6 | 33.4 | 108.5 | 9.5 | 278.2 | 5.7 | 3.8 | 857.7 |
| **1978** | 129.5 | 20.7 | 60.8 | 341.7 | 132.8 | 4.4 | 75.6 | 186.6 | 13.3 | 380.6 | 4.5 | 2.4 | 1352.9 |
| **1980** | 55.5 | 10.3 | 25.3 | 206.5 | 52.4 | 2.3 | 29.3 | 66.3 | 5.9 | 151.7 | 6.4 | 2.1 | 613.9 |
| **1987** | 30.5 | 5.8 | 12.8 | 60.1 | 26.2 | 1.6 | 8.7 | 8.2 | 1.9 | 68.1 | 7.9 | 0.2 | 232.1 |
| **2105** | 163.1 | 28.5 | 104.7 | 269.7 | 264.7 | 9.5 | 117.8 | 49.9 | 22.7 | 766.5 | 18.8 | 5.2 | 1821.2 |
| **2106** | 221.6 | 49.7 | 131.3 | 464.4 | 282.8 | 10.9 | 85.4 | 61.1 | 31.6 | 920.3 | 19.5 | 9.5 | 2288 |
| **2113** | 107.2 | 23.5 | 71.8 | 209.4 | 135.6 | 6.4 | 50.7 | 30.3 | 16.4 | 509.2 | 20.4 | 4.8 | 1185.7 |

| **Table S4:** The individual sesquiterpenes (ng mg^-1^ fresh weight) in 30 families of *Picea glauca* foliage from Calling Lake, Alberta. | | | | | | | | | | | |
| --- | --- | --- | --- | --- | --- | --- | --- | --- | --- | --- | --- |
| **Family** | **α-Cadinene** | **α -Cadinol** | **Cubebol** | **Cubenene** | **Germacrene D-4-ol** | **Germacrene-D** | **γ-Cadinene** | **Σ-Cadinene** | **tau-cadinol** | **Total** |  |
| **128** | 0 | 0.2 | 42.8 | 0.3 | 274 | 0.3 | 2.3 | 3.9 | 1 | 325 |  |
| **133** | 0 | 0.9 | 117.7 | 2.9 | 634.1 | 0.4 | 9.1 | 13.4 | 5.9 | 784.4 |  |
| **143** | 0 | 0 | 91.5 | 0.9 | 439 | 0 | 6.4 | 8.8 | 5.1 | 551 |  |
| **148** | 0 | 5 | 210 | 6 | 1083 | 0.4 | 17.9 | 27.1 | 13.5 | 1363 |  |
| **156** | 0 | 9.136 | 323.6 | 7.36 | 2016 | 0.811 | 22.39 | 33.73 | 18.03 | 2431 |  |
| **157** | 0 | 2.4 | 126 | 2.57 | 783 | 0.3 | 9 | 14.1 | 6.6 | 944 |  |
| **158** | 0 | 0.8 | 171 | 1.5 | 944 | 2.8 | 0.9 | 3.2 | 2.4 | 1126 |  |
| **170** | 1.9 | 0 | 306.6 | 2.9 | 1947 | 6.3 | 3 | 9.2 | 2.4 | 2279 |  |
| **176** | 2.2 | 0 | 356 | 8.2 | 2447 | 10.1 | 4.4 | 13.1 | 5.1 | 2846 |  |
| **178** | 2.35 | 0 | 159 | 1.89 | 1056 | 3.9 | 3.9 | 9.5 | 2.1 | 1239 |  |
| **180** | 0.9 | 0 | 148 | 1.5 | 949 | 3 | 2.4 | 6.5 | 3.9 | 1115 |  |
| **183** | 0 | 0.4 | 93.2 | 0.7 | 643 | 1.9 | 1.8 | 3.5 | 0.8 | 745 |  |
| **188** | 0 | 2.4 | 207 | 0.5 | 1677 | 5.6 | 4.4 | 9.3 | 4.8 | 1911 |  |
| **190** | 0 | 0.8 | 164 | 0.3 | 1222 | 4.6 | 3.7 | 7.2 | 3.1 | 1406 |  |
| **195** | 1.5 | 1.1 | 168 | 0.5 | 1349 | 5.9 | 4.5 | 12.6 | 3.1 | 1546 |  |
| **201** | 0 | 2.1 | 183 | 0.3 | 1489 | 4.8 | 5.3 | 9.1 | 6.5 | 1701 |  |
| **203** | 0 | 1.8 | 215.7 | 0.6 | 1758 | 5.9 | 9.1 | 15.4 | 6.6 | 2013 |  |
| **1580** | 0 | 1.1 | 191 | 0.1 | 1637 | 4.8 | 8.8 | 12.7 | 7.8 | 1863 |  |
| **1917** | 1.5 | 0.7 | 176.1 | 0.1 | 1380 | 5.2 | 10.9 | 14 | 9.4 | 1598 |  |
| **1918** | 2.8 | 0.4 | 93.6 | 0.1 | 576.2 | 2.1 | 7.8 | 7.9 | 5.6 | 696.4 |  |
| **1924** | 0.7 | 0.4 | 62.9 | 0 | 394.9 | 2.2 | 5.1 | 8.4 | 2.4 | 477 |  |
| **1951** | 1.9 | 1.3 | 108.3 | 0.6 | 569.2 | 3 | 10.4 | 12.9 | 5.9 | 713.6 |  |
| **1952** | 1.6 | 3.2 | 213.9 | 0.1 | 1389 | 6 | 21 | 28.2 | 17.2 | 1681 |  |
| **1976** | 0 | 0.2 | 99.9 | 0.1 | 661.7 | 1.9 | 8 | 9.7 | 5.6 | 787.2 |  |
| **1978** | 0 | 0.9 | 139.6 | 0.7 | 1042 | 4.3 | 9.7 | 16.9 | 7.2 | 1221 |  |
| **1980** | 0 | 0.9 | 139.6 | 0.7 | 1042 | 4.3 | 9.7 | 16.9 | 7.2 | 1221 |  |
| **1987** | 0 | 1.6 | 150.8 | 0.4 | 1100.8 | 6.3 | 8.8 | 12.7 | 5.3 | 1286.6 |  |
| **2105** | 1.2 | 0.7 | 93.7 | 0 | 712.1 | 2.8 | 5.6 | 12 | 2.3 | 830.5 |  |
| **2106** | 1 | 1.4 | 190.1 | 0 | 1598.4 | 7.6 | 8.7 | 14.9 | 6.8 | 1828.9 |  |
| **2113** | 0 | 2.1 | 131.7 | 0.3 | 1081.8 | 6.1 | 8.3 | 16.4 | 6.4 | 1253.1 |  |

| **Table S5:** The individual sesquiterpenes (ng mg^-1^ fresh weight) in 30 families of *Picea glauca* foliage from Carson Lake, Alberta. | | | | | | | | | | | |
| --- | --- | --- | --- | --- | --- | --- | --- | --- | --- | --- | --- |
| **Family** | **α-Cadinene** | **α -Cadinol** | **Cubebol** | **Cubenene** | **Germacrene D-4-ol** | **Germacrene-D** | **γ-Cadinene** | **Σ-Cadinene** | **tau-cadinol** | **Total** |  |
| **128** | 11 | 0.5 | 192.2 | 1.8 | 834.3 | 3 | 6.4 | 9 | 3.4 | 1061.6 |  |
| **133** | 29.5 | 3.6 | 315 | 7.6 | 1996.5 | 8.4 | 21.4 | 32.6 | 15.3 | 2429.9 |  |
| **143** | 3.1 | 0.8 | 84.6 | 1.7 | 562.8 | 2.5 | 4.9 | 10 | 3.2 | 673.6 |  |
| **148** | 22.1 | 1.3 | 173.7 | 5 | 821.3 | 5.9 | 14.8 | 26 | 9.2 | 1079.2 |  |
| **156** | 79.7 | 11 | 665.5 | 15.7 | 3200.5 | 17 | 38.6 | 47.4 | 28.4 | 4103.7 |  |
| **157** | 11.8 | 0.9 | 94.1 | 2 | 532.5 | 2.8 | 7.4 | 10.8 | 3.9 | 666 |  |
| **158** | 17.5 | 2.2 | 164.7 | 3.6 | 1034.1 | 4.4 | 10.3 | 15.4 | 5.3 | 1257.6 |  |
| **170** | 15.7 | 1.4.0 | 93.2 | 3 | 579.4 | 2.9 | 8.5 | 11.3 | 5.8 | 721.3 |  |
| **176** | 64.4 | 5.6 | 357.1 | 12.1 | 2648.3 | 14.8 | 34.3 | 44.9 | 26.9 | 3208.4 |  |
| **178** | 0.8 | 0 | 16.1 | 0 | 95.5 | 0.1 | 1.6 | 2.7 | 0.4 | 117.3 |  |
| **180** | 20 | 2.6 | 124.5 | 5.3 | 886.3 | 5.4 | 12.1 | 15.8 | 8.8 | 1080.8 |  |
| **183** | 14 | 2.6 | 68.6 | 3.7 | 439.6 | 4.8 | 5.4 | 10.5 | 4.7 | 553.9 |  |
| **188** | 38.9 | 5 | 81.9 | 9.5 | 532.7 | 7.4 | 19.1 | 34.8 | 4.1 | 733.3 |  |
| **190** | 38.7 | 4.2 | 71.4 | 8.5 | 483.2 | 11.5 | 30.5 | 46.1 | 3.8 | 698 |  |
| **195** | 33.9 | 4.7 | 153.3 | 15.3 | 1202.5 | 18.7 | 20.7 | 38.8 | 9.5 | 1497.4 |  |
| **201** | 20.5 | 2.9 | 142.7 | 4.8 | 1073.6 | 5.8 | 12.6 | 19.4 | 14.3 | 1296.5 |  |
| **203** | 35.5 | 3.4 | 249.3 | 8.8 | 1869.5 | 9.9 | 21.5 | 36.2 | 24.5 | 2258.6 |  |
| **1580** | 25.5 | 0.9 | 154.6 | 5.2 | 1234.9 | 5.5 | 15.4 | 20.2 | 15.6 | 1477.9 |  |
| **1917** | 35.4 | 3 | 215.3 | 7.2 | 1651.3 | 9.5 | 20 | 29.1 | 26 | 1996.8 |  |
| **1918** | 10 | 1.5 | 79.3 | 2.2 | 629.9 | 4 | 6.6 | 10.4 | 6.8 | 750.8 |  |
| **1924** | 6.4 | 0.3 | 57.2 | 0.7 | 419.2 | 2.4 | 4.7 | 7.4 | 2.9 | 501.3 |  |
| **1951** | 5.9 | 0 | 48.9 | 0.8 | 295.5 | 2 | 4.1 | 6.3 | 2.5 | 366.1 |  |
| **1952** | 35 | 4.3 | 188.5 | 8.3 | 976.5 | 4.5 | 19 | 30.5 | 19.2 | 1285.8 |  |
| **1976** | 6.9 | 0 | 70.6 | 1 | 430.3 | 3.3 | 5.4 | 8.6 | 1.9 | 528.1 |  |
| **1978** | 21.3 | 2 | 181.8 | 4.6 | 1298.7 | 7.3 | 14.2 | 23 | 13.2 | 1566.2 |  |
| **1980** | 6.1 | 0.9 | 70.8 | 2.1 | 442 | 2 | 5.7 | 8.8 | 4.9 | 543.2 |  |
| **1987** | 0 | 0 | 31.2 | 0.8 | 196.2 | 0 | 2.2 | 3.1 | 0.8 | 234.4 |  |
| **2105** | 0 | 1.7 | 158.3 | 3.3 | 1190.1 | 0.1 | 10.9 | 17.4 | 9.2 | 1391.1 |  |
| **2106** | 1 | 3.8 | 310.8 | 8.3 | 2433.3 | 1.2 | 20.8 | 30.5 | 16.7 | 2826.4 |  |
| **2113** | 0 | 2.1 | 148.2 | 2.4 | 1023.9 | 0.4 | 7.8 | 11.9 | 8.2 | 1204.8 |  |

**Table S6.** Secondary metabolite profiles were identified in the mycelium of ten endophytic fungi of *Picea glauca* foliage.

| Secondary metabolites | Chemical formula | Class |
| --- | --- | --- |
| Ergosterol | C_28_H_44_O | Sterol |
| Farnesol | C_15_H_26_O | Sesquiterpene |
| Squalene | C₃₀H₅₀ | Triterpene |
| 4-Methyl-1,6-heptadien-4-ol | C_8_H_14_O | Oxygenated monoterpene |
| 9,12-octadecadienoic acid | C_18_H_32_O_2_ | Fatty acid |
| 1-Octanol,2-butyl | C_12_H_26_O | Fatty aldehyde |
| oxalic acid_6-ethyloct-3-yl | C_19_H_36_O_4_ | Glycol |
| 1-Decanol, 2-hexyl | C_16_H_34_O | Glycol |
| n-hexadecanoic acid | C_16_H_32_O_2_ | Fatty acid |
| 2-Dodecenal | C_12_H_22_O | Fatty aldehyde |
| 9-Octadecenal | C_18_H_34_O | Fatty aldehyde |

| Endophytic fungi | Ergosterol | Farnesol | Squalene | 4-Methyl-1,6-heptadien-4-ol | 9,12-Octadecadienoic  acid | 1-Octanol,2-butyl | oxalic acid_6-ethyloct-3-yl | 1-Decanol,2-hexyl | n-hexadecanoic acid | Dodecenal | 9-Octadecenal | Total |
| --- | --- | --- | --- | --- | --- | --- | --- | --- | --- | --- | --- | --- |
| *Chalara*_1 | 23889.5 | 25.1 | 49.8 | 14.6 | 20729.9 | 754.4 | 58.2 | 6.6 | 6880.0 | 1 | 27.3 | 52436.5 |
| *Cladosporium*_2 | 7987.1 | 16.0 | 136.7 | 28.4 | 5348.2 | 1039.0 | 56.5 | 11.2 | 12624.0 | 93.1 | 123.4 | 27463.2 |
| *Cladosporium*_2 | 10777.2 | 17.3 | 164.3 | 18.6 | 5234.5 | 52.1 | 90.5 | 15.6 | 4415.5 | 1 | 92.7 | 20879.4 |
| *Didymella*_1 | 18178.6 | 22.9 | 29.6 | 16.1 | 241.3 | 955.2 | 28.5 | 28.7 | 64.8 | 11.8 | 20.9 | 19598.4 |
| *Dothideomycetes*_1 | 11185.0 | 14.2 | 50.5 | 56.8 | 6842.7 | 299.4 | 73.4 | 22.6 | 2045.1 | 12.7 | 43.6 | 20645.9 |
| *Chalara*_1 | 23889.5 | 25.1 | 49.8 | 14.6 | 20729.9 | 754.4 | 58.2 | 6.6 | 6880.0 | 1 | 27.3 | 52436.5 |
| *Geopyxis*_2 | 5150.9 | 6.3 | 20.3 | 5.4 | 8545.9 | 278.2 | 7.9 | 5.6 | 2682.7 | 14.4 | 0.0 | 16718.6 |
| *Geopyxis*_3 | 6343.3 | 14.9 | 373.8 | 25.9 | 327.7 | 883.2 | 66.8 | 10.9 | 516.4 | 41.4 | 17.5 | 8621.8 |
| *Geopyxis*_4 | 21639.4 | 22.8 | 1143.0 | 25.3 | 1318.8 | 1170.0 | 53.3 | 14.9 | 232.2 | 10.4 | 83.8 | 25714.0 |
| *Pezizales*_1 | 11503.4 | 21.5 | 303.4 | 37.9 | 125.2 | 525.0 | 52.4 | 21.6 | 77.4 | 28.9 | 47.1 | 12743.9 |

**Table S7.** Concentrations of individual mycelium metabolite (ng mg^-^1 DE dry weight) in 10 endophytic fungi of *Picea glauca* foliage.

**Table S8.** Endophytic fungal volatile organic compounds were identified from ten endophytic fungi of *Picea glauca* foliage.

| Fungal VOCs | Chemical formula | Class |
| --- | --- | --- |
| α-Pinene | C_10_H_16_ | Monoterpene |
| Camphene | C_10_H_16_ | Monoterpene |
| *p*-Cymene | C_10_H_16_ | Monoterpene |
| β-Pinene | C_10_H_16_ | Monoterpene |
| 3-Carene | C_10_H_16_ | Monoterpene |
| β-Phellandrene | C_10_H_16_ | Monoterpene |
| 2,5-Dimethyl-1,5-hexadien-3-ol | C_8_H_14_O | Oxygenated monoterpene |
| 2-Cyclopenten-1-one | C_5_H_6_O | Ketone |
| 2,4-Dimethyl-1-heptane | C_9_H_18_ | Alkane |
| 2,4,6,8-Tetramethyl-1-undecane | C_15_H_30_ | Alkane |
| Butanal-3 methyl | C_5_H_10_O | Aldehyde |
| Hexyl octyl ether | C_14_H_30_O | Ether |
| Pentanal | C_5_H_10_O | Aldehyde |

**Table S9.** Concentrations of individual volatile organic compounds (ng mg^-^1 dry weight) in 10 endophytic fungi of *Picea glauca* foliage. ND refers to not detected.

| Family | α-Pinene | Camphene | p-Cymene | β-Pinene | 3-Carene | β-Phellandrene | 2,4-Dimethyl-1-heptane | 2,5-Dimethyl-1,5-hexadien-3-ol | 2-cyclopenten-1-one | 2,4,6,8-Tetramethyl-1-undecane | butanal-3 methyl | Hexyl octyl ether | Pentanal | Total |
| --- | --- | --- | --- | --- | --- | --- | --- | --- | --- | --- | --- | --- | --- | --- |
| *Geopyxis*_1 | 8.1 | 1.2 | 1.4 | 17.6 | 10.4 | 18.5 | 5.3 | 6.1 | 3.8 | 0.5 | 2.2 | 10.5 | 0.4 | 85.8 |
| *Chalara* sp. | ND | ND | ND | ND | ND | ND | 6.4 | 5.9 | ND | 0.7 | 2.8 | 9.0 | 0.6 | 25.4 |
| *Cladosporium*_1 | ND | ND | ND | ND | ND | ND | 6.4 | 8.4 | ND | 3.7 | 3.2 | 11.5 | 0.6 | 33.6 |
| *Geopyxis*_2 | ND | ND | ND | ND | ND | ND | 5.3 | 5.9 | ND | 0.5 | 1.4 | 7.4 | 0.5 | 21.2 |
| *Dothideomycetes*_1 | 7.8 | 1.1 | 15.8 | 13.6 | 21.8 | 7.3 | 5.8 | 6.2 | 7.7 | 0.5 | 2.7 | 11.7 | 0.4 | 102.4 |
| *Pezizales*_1 | ND | ND | ND | ND | ND | ND | 5.4 | 7.1 | ND | 0.5 | 2.3 | 10.6 | 0.5 | 26.4 |
| *Cladosporium*_2 | ND | ND | ND | ND | ND | ND | 5.6 | 9.9 | ND | 1.5 | 3.4 | 12.4 | 0.5 | 33.3 |
| *Geopyxis*_4 | 4.4 | 0.54 | 1.4 | 4.2 | 5.8 | 9.6 | 4.2 | 3.4 | 4.6 | 5.3 | 1.0 | 14.8 | 0.3 | 59.5 |
| *Geopyxis*_3 | ND | ND | ND | ND | ND | ND | 3.2 | 3.4 | ND | 6.8 | 1.3 | 10.7 | 0.3 | 25.7 |
| *Didymella*_1 | ND | ND | ND | ND | ND | ND | 3.3 | 3.3 | ND | 8.1 | 1.1 | 10.0 | 0.2 | 25.9 |
|  |  |  |  |  |  |  |  |  |  |  |  |  |  |  |

**Table S10.** The individual monoterpenes (ng mg^-1^) in 30 inoculated and control families/genotypes of *Picea glauca* seedling foliage.

| **Group** | **Families** | α-Pinene | Camphene | β-Pinene | β-Myrcene | Limonene | 3-Carene | Terpinolene | Camphor | α-Terpineol | α- Terpinene | Borneol | endo-Borneol | Linalool | Sabinene hydrate | Bornyl acetate | Geranyl acetate | β-Citral | β-Phellandrene | Verbenone | Eucalyptol | Tricyclene | Sabinene | Camphene hydrate | Citronellol | Citronellol acetate | Total monoterpenes |
| --- | --- | --- | --- | --- | --- | --- | --- | --- | --- | --- | --- | --- | --- | --- | --- | --- | --- | --- | --- | --- | --- | --- | --- | --- | --- | --- | --- |
| **Inoculated** | 128 | 665 | 1011 | 121 | 910 | 766 | 5 | 129 | 2126 | 21 | 1 | 85 | 3 | 12 | 16 | 2454 | 64 | 4 | 11 | 8 | 23 | 81 | 18 | 2 | 60 | 16 | 8612 |
| **Control** | 128 | 152 | 236 | 30 | 243 | 178 | 0 | 28 | 252 | 4 | 0 | 10 | 0 | 3 | 2 | 852 | 90 | 4 | 3 | 0 | 3 | 18 | 0 | 0 | 26 | 10 | 2146 |
| **Inoculated** | 133 | 605 | 933 | 129 | 655 | 664 | 2 | 114 | 2244 | 21 | 0 | 57 | 3 | 9 | 16 | 1336 | 148 | 8 | 12 | 5 | 12 | 74 | 4 | 3 | 47 | 22 | 7119 |
| **Control** | 133 | 608 | 938 | 115 | 844 | 588 | 2 | 112 | 2214 | 16 | 0 | 53 | 2 | 11 | 15 | 1417 | 172 | 9 | 16 | 4 | 11 | 74 | 3 | 2 | 49 | 24 | 7301 |
| **Inoculated** | 143 | 546 | 863 | 111 | 855 | 1072 | 2 | 111 | 2305 | 19 | 1 | 45 | 5 | 15 | 15 | 1696 | 43 | 12 | 65 | 6 | 16 | 65 | 3 | 2 | 74 | 12 | 7957 |
| **Control** | 143 | 578 | 891 | 110 | 905 | 882 | 2 | 106 | 2241 | 15 | 0 | 41 | 3 | 15 | 15 | 1792 | 85 | 5 | 14 | 5 | 10 | 68 | 3 | 2 | 109 | 20 | 7917 |
| **Inoculated** | 148 | 652 | 1023 | 125 | 1140 | 744 | 3 | 112 | 2425 | 17 | 1 | 47 | 4 | 10 | 16 | 2291 | 167 | 28 | 18 | 11 | 11 | 80 | 3 | 3 | 44 | 20 | 8994 |
| **Control** | 148 | 430 | 665 | 85 | 645 | 450 | 4 | 82 | 1287 | 10 | 0 | 28 | 2 | 11 | 11 | 2049 | 154 | 16 | 11 | 5 | 5 | 48 | 2 | 1 | 52 | 21 | 6074 |
| **Inoculated** | 156 | 787 | 1192 | 167 | 884 | 1131 | 5 | 150 | 3518 | 38 | 1 | 122 | 4 | 25 | 28 | 2848 | 63 | 5 | 33 | 15 | 45 | 109 | 7 | 7 | 74 | 17 | 11275 |
| **Control** | 156 | 611 | 1001 | 138 | 745 | 917 | 2 | 143 | 3295 | 34 | 1 | 79 | 1 | 17 | 25 | 1751 | 90 | 7 | 47 | 11 | 25 | 75 | 5 | 3 | 85 | 16 | 9124 |
| **Inoculated** | 157 | 560 | 880 | 117 | 671 | 871 | 2 | 107 | 1972 | 21 | 0 | 54 | 0 | 11 | 17 | 2163 | 50 | 5 | 34 | 5 | 16 | 65 | 3 | 2 | 88 | 20 | 7734 |
| **Control** | 157 | 350 | 568 | 69 | 596 | 555 | 0 | 69 | 1552 | 15 | 0 | 26 | 0 | 8 | 12 | 1246 | 39 | 2 | 7 | 3 | 20 | 41 | 2 | 1 | 78 | 21 | 5281 |
| **Inoculated** | 158 | 281 | 455 | 53 | 304 | 400 | 1 | 51 | 1124 | 9 | 0 | 16 | 2 | 5 | 9 | 1178 | 119 | 3 | 7 | 3 | 7 | 34 | 2 | 0 | 28 | 25 | 4116 |
| **Control** | 158 | 326 | 507 | 63 | 347 | 537 | 1 | 59 | 957 | 13 | 0 | 16 | 1 | 5 | 9 | 1553 | 12 | 4 | 69 | 2 | 18 | 38 | 3 | 0 | 17 | 6 | 4563 |
| **Inoculated** | 170 | 786 | 1226 | 174 | 898 | 975 | 3 | 164 | 2962 | 25 | 1 | 52 | 6 | 13 | 27 | 3135 | 240 | 23 | 38 | 4 | 21 | 98 | 5 | 6 | 65 | 36 | 10983 |
| **Control** | 170 | 460 | 756 | 98 | 602 | 764 | 2 | 105 | 1513 | 18 | 0 | 29 | 4 | 8 | 16 | 2530 | 189 | 11 | 46 | 4 | 17 | 59 | 3 | 3 | 51 | 49 | 7337 |
| **Inoculated** | 176 | 667 | 1023 | 138 | 516 | 986 | 7 | 110 | 2494 | 24 | 1 | 49 | 3 | 24 | 21 | 1976 | 191 | 12 | 22 | 7 | 21 | 80 | 4 | 2 | 141 | 45 | 8565 |
| **Control** | 176 | 376 | 626 | 100 | 376 | 539 | 4 | 68 | 1240 | 9 | 0 | 17 | 1 | 15 | 9 | 1221 | 102 | 6 | 21 | 5 | 0 | 46 | 2 | 1 | 111 | 19 | 4913 |
| **Inoculated** | 178 | 803 | 1275 | 163 | 1032 | 1394 | 3 | 167 | 3383 | 23 | 1 | 71 | 1 | 16 | 24 | 2604 | 113 | 6 | 17 | 16 | 8 | 97 | 4 | 3 | 64 | 30 | 11315 |
| **Control** | 178 | 56 | 415 | 180 | 905 | 449 | 3 | 173 | 1698 | 24 | 1 | 76 | 1 | 19 | 27 | 3012 | 176 | 10 | 67 | 11 | 6 | 108 | 4 | 3 | 11 | 25 | 7460 |
| **Inoculated** | 180 | 759 | 1180 | 172 | 848 | 1089 | 3 | 156 | 3087 | 45 | 1 | 57 | 3 | 22 | 29 | 2466 | 73 | 14 | 17 | 10 | 52 | 91 | 6 | 3 | 119 | 22 | 10322 |
| **Control** | 180 | 293 | 456 | 58 | 338 | 426 | 4 | 48 | 763 | 8 | 0 | 17 | 2 | 5 | 7 | 1383 | 61 | 10 | 8 | 1 | 8 | 34 | 2 | 0 | 39 | 14 | 3985 |
| **Inoculated** | 183 | 610 | 951 | 129 | 560 | 965 | 2 | 108 | 2301 | 21 | 1 | 53 | 4 | 13 | 17 | 2056 | 29 | 2 | 18 | 6 | 18 | 76 | 4 | 3 | 12 | 11 | 7970 |
| **Control** | 183 | 361 | 606 | 75 | 233 | 495 | 0 | 72 | 1321 | 10 | 0 | 42 | 2 | 8 | 10 | 1576 | 76 | 4 | 11 | 3 | 2 | 47 | 2 | 1 | 8 | 19 | 4984 |
| **Inoculated** | 188 | 379 | 610 | 69 | 595 | 608 | 1 | 63 | 1199 | 9 | 0 | 59 | 1 | 6 | 10 | 1762 | 101 | 40 | 17 | 3 | 7 | 53 | 2 | 9 | 49 | 15 | 5666 |
| **Control** | 188 | 403 | 638 | 73 | 1022 | 755 | 1 | 47 | 1517 | 9 | 0 | 58 | 3 | 9 | 10 | 1421 | 156 | 13 | 15 | 8 | 6 | 53 | 2 | 5 | 88 | 20 | 6335 |
| **Inoculated** | 190 | 719 | 1091 | 174 | 1056 | 1013 | 9 | 131 | 2710 | 28 | 1 | 57 | 3 | 17 | 23 | 2675 | 49 | 8 | 43 | 9 | 35 | 86 | 6 | 6 | 58 | 14 | 10019 |
| **Control** | 190 | 474 | 771 | 96 | 549 | 535 | 3 | 95 | 1443 | 16 | 1 | 27 | 3 | 10 | 13 | 2315 | 76 | 9 | 19 | 6 | 10 | 61 | 3 | 2 | 58 | 20 | 6610 |
| **Inoculated** | 195 | 627 | 1027 | 127 | 847 | 949 | 2 | 129 | 2760 | 20 | 1 | 46 | 3 | 10 | 20 | 1877 | 111 | 3 | 12 | 6 | 26 | 79 | 4 | 2 | 63 | 31 | 8782 |
| **Control** | 195 | 448 | 723 | 93 | 665 | 687 | 1 | 90 | 1734 | 12 | 0 | 37 | 1 | 9 | 13 | 1616 | 96 | 2 | 9 | 5 | 8 | 55 | 3 | 2 | 73 | 41 | 6422 |
| **Inoculated** | 201 | 501 | 789 | 95 | 670 | 651 | 3 | 96 | 1994 | 11 | 1 | 57 | 0 | 10 | 14 | 1773 | 94 | 10 | 9 | 4 | 0 | 60 | 2 | 4 | 71 | 24 | 6941 |
| **Control** | 201 | 556 | 856 | 122 | 597 | 684 | 14 | 111 | 1750 | 14 | 1 | 35 | 3 | 9 | 14 | 2352 | 51 | 13 | 13 | 4 | 7 | 65 | 3 | 3 | 63 | 19 | 7360 |
| **Inoculated** | 203 | 409 | 643 | 82 | 442 | 698 | 1 | 82 | 1531 | 11 | 0 | 40 | 3 | 7 | 12 | 1608 | 36 | 1 | 8 | 5 | 6 | 47 | 2 | 1 | 52 | 15 | 5742 |
| **Control** | 203 | 186 | 290 | 36 | 371 | 354 | 0 | 31 | 708 | 5 | 0 | 14 | 1 | 4 | 5 | 689 | 23 | 0 | 8 | 2 | 3 | 22 | 1 | 0 | 9 | 10 | 2771 |
| **Inoculated** | 1586 | 472 | 675 | 117 | 818 | 568 | 4 | 69 | 1100 | 15 | 1 | 36 | 2 | 10 | 10 | 1829 | 142 | 25 | 17 | 3 | 14 | 51 | 3 | 2 | 80 | 31 | 6092 |
| **Control** | 1586 | 217 | 333 | 39 | 456 | 183 | 3 | 32 | 285 | 7 | 0 | 15 | 0 | 3 | 3 | 995 | 113 | 11 | 4 | 1 | 5 | 27 | 1 | 2 | 31 | 24 | 2791 |
| **Inoculated** | 1917 | 340 | 570 | 84 | 557 | 442 | 3 | 91 | 1279 | 13 | 1 | 44 | 2 | 8 | 12 | 2099 | 104 | 6 | 19 | 7 | 7 | 48 | 2 | 4 | 34 | 22 | 5795 |
| **Control** | 1917 | 223 | 388 | 49 | 333 | 281 | 1 | 49 | 700 | 8 | 0 | 21 | 1 | 5 | 7 | 1382 | 76 | 4 | 6 | 4 | 9 | 29 | 2 | 1 | 20 | 27 | 3625 |
| **Inoculated** | 1918 | 565 | 856 | 117 | 1128 | 592 | 1 | 86 | 1815 | 13 | 1 | 47 | 2 | 8 | 12 | 1828 | 363 | 14 | 18 | 4 | 7 | 75 | 3 | 9 | 94 | 67 | 7727 |
| **Control** | 1918 | 236 | 343 | 56 | 390 | 324 | 0 | 33 | 396 | 5 | 1 | 15 | 1 | 5 | 5 | 1117 | 128 | 4 | 24 | 2 | 3 | 31 | 1 | 4 | 42 | 35 | 3200 |
| **Inoculated** | 1924 | 314 | 487 | 65 | 567 | 494 | 1 | 58 | 1073 | 8 | 0 | 21 | 1 | 9 | 9 | 1298 | 43 | 4 | 12 | 1 | 0 | 38 | 1 | 1 | 50 | 22 | 4576 |
| **Control** | 1924 | 74 | 109 | 14 | 198 | 117 | 0 | 13 | 65 | 1 | 0 | 2 | 0 | 1 | 1 | 502 | 87 | 0 | 17 | 0 | 1 | 8 | 0 | 0 | 24 | 33 | 1268 |
| **Inoculated** | 1951 | 333 | 505 | 78 | 494 | 445 | 6 | 54 | 1098 | 19 | 0 | 24 | 1 | 8 | 10 | 1296 | 42 | 3 | 13 | 3 | 36 | 39 | 3 | 2 | 42 | 14 | 4567 |
| **Control** | 1951 | 305 | 463 | 66 | 494 | 391 | 4 | 48 | 783 | 19 | 0 | 42 | 1 | 9 | 10 | 1448 | 63 | 4 | 14 | 1 | 35 | 42 | 3 | 5 | 13 | 13 | 4277 |
| **Inoculated** | 1952 | 446 | 703 | 87 | 834 | 807 | 2 | 74 | 1768 | 21 | 1 | 62 | 5 | 12 | 15 | 1938 | 48 | 6 | 63 | 6 | 41 | 52 | 4 | 1 | 79 | 25 | 7099 |
| **Control** | 1952 | 266 | 430 | 49 | 424 | 449 | 0 | 42 | 839 | 13 | 0 | 15 | 2 | 8 | 7 | 1206 | 59 | 2 | 6 | 2 | 23 | 33 | 2 | 0 | 90 | 36 | 4002 |
| **Inoculated** | 1976 | 415 | 643 | 96 | 555 | 561 | 2 | 77 | 1195 | 10 | 1 | 36 | 2 | 10 | 10 | 1951 | 69 | 13 | 8 | 4 | 6 | 49 | 2 | 2 | 74 | 21 | 5815 |
| **Control** | 1976 | 476 | 776 | 99 | 679 | 703 | 1 | 92 | 1346 | 11 | 1 | 45 | 3 | 11 | 11 | 2085 | 43 | 18 | 11 | 4 | 2 | 66 | 2 | 3 | 89 | 18 | 6594 |
| **Inoculated** | 1978 | 369 | 588 | 106 | 469 | 779 | 2 | 57 | 1508 | 11 | 0 | 35 | 3 | 10 | 11 | 1629 | 34 | 4 | 12 | 4 | 17 | 45 | 2 | 0 | 65 | 17 | 5775 |
| **Control** | 1978 | 154 | 440 | 95 | 205 | 153 | 1 | 96 | 602 | 13 | 1 | 60 | 3 | 10 | 14 | 2096 | 44 | 12 | 14 | 5 | 24 | 61 | 3 | 2 | 71 | 13 | 4192 |
| **Inoculated** | 1980 | 381 | 583 | 85 | 481 | 589 | 1 | 70 | 1331 | 12 | 1 | 48 | 2 | 7 | 13 | 2140 | 29 | 1 | 9 | 5 | 14 | 47 | 2 | 2 | 49 | 14 | 5916 |
| **Control** | 1980 | 270 | 422 | 50 | 389 | 335 | 1 | 43 | 511 | 5 | 0 | 19 | 2 | 5 | 6 | 1828 | 99 | 1 | 5 | 2 | 2 | 35 | 1 | 0 | 36 | 22 | 4088 |
| **Inoculated** | 1986 | 662 | 976 | 145 | 850 | 1199 | 2 | 110 | 2609 | 24 | 1 | 70 | 3 | 13 | 20 | 1988 | 97 | 28 | 69 | 4 | 42 | 76 | 4 | 3 | 82 | 27 | 9102 |
| **Control** | 1986 | 379 | 574 | 72 | 570 | 418 | 1 | 59 | 976 | 11 | 1 | 26 | 2 | 5 | 8 | 1603 | 206 | 21 | 9 | 2 | 19 | 42 | 2 | 0 | 12 | 12 | 5030 |
| **Inoculated** | 2105 | 677 | 1022 | 139 | 1352 | 1280 | 7 | 124 | 2679 | 20 | 1 | 73 | 6 | 18 | 19 | 1757 | 144 | 19 | 28 | 7 | 21 | 87 | 5 | 8 | 125 | 21 | 9639 |
| **Control** | 2105 | 321 | 531 | 76 | 709 | 631 | 1 | 59 | 1201 | 10 | 0 | 42 | 5 | 10 | 11 | 1092 | 268 | 14 | 14 | 5 | 11 | 43 | 3 | 4 | 97 | 17 | 5174 |
| **Inoculated** | 2106 | 570 | 905 | 160 | 778 | 826 | 2 | 137 | 2311 | 28 | 1 | 84 | 7 | 14 | 19 | 2179 | 80 | 20 | 16 | 18 | 20 | 78 | 4 | 7 | 72 | 23 | 8359 |
| **Control** | 2106 | 355 | 580 | 99 | 357 | 501 | 1 | 90 | 1203 | 15 | 0 | 35 | 4 | 8 | 13 | 1711 | 142 | 7 | 9 | 6 | 13 | 46 | 3 | 3 | 73 | 19 | 5292 |
| **Inoculated** | 2113 | 536 | 782 | 114 | 852 | 748 | 2 | 104 | 1770 | 18 | 1 | 53 | 4 | 12 | 16 | 2208 | 92 | 8 | 12 | 10 | 32 | 66 | 5 | 3 | 47 | 11 | 7503 |
| **Control** | 2113 | 333 | 503 | 73 | 856 | 469 | 1 | 64 | 1006 | 7 | 0 | 28 | 2 | 8 | 9 | 1603 | 80 | 7 | 8 | 6 | 2 | 40 | 2 | 2 | 52 | 25 | 5187 |

**Table S11:** The individual sesquiterpenes (ng mg^-1^) in 30 inoculated and control families/genotypes of *Picea glauca* seedling foliage.

| **Group** | **Families** | **Germacrene D-4-ol** | **Germacrene D** | **β-Elemene** | **γ-Cadinene** | **Oplopanone** | **α-Cadinene** | **Total sesquiterpene** |
| --- | --- | --- | --- | --- | --- | --- | --- | --- |
| **Inoculated** | 128 | 187.9 | 22.3 | 6.3 | 4.11 | 32.9 | 17.1 | 270.8 |
| **Control** | 128 | 49.9 | 15.4 | 0.2 | 0 | 4.78 | 2.2 | 72.6 |
| **Inoculated** | 133 | 180.7 | 49.5 | 5.1 | 1.8 | 19.7 | 10.6 | 267.5 |
| **Control** | 133 | 194.3 | 31.5 | 5.5 | 1.85 | 17.8 | 9.6 | 260.6 |
| **Inoculated** | 143 | 189.8 | 19.3 | 6.4 | 1.75 | 20.8 | 17.8 | 256.1 |
| **Control** | 143 | 151.5 | 11.8 | 5.3 | 1.71 | 16.1 | 7.77 | 194.2 |
| **Inoculated** | 148 | 162.4 | 13.3 | 3.6 | 2.1 | 18 | 8.9 | 208.4 |
| **Control** | 148 | 114.5 | 16.9 | 3.3 | 1.2 | 15 | 6.71 | 157.7 |
| **Inoculated** | 156 | 274.4 | 19.2 | 8.1 | 4.0 | 29.5 | 16.9 | 352.3 |
| **Control** | 156 | 257.6 | 25.4 | 6.9 | 3.8 | 19.3 | 15.6 | 328.7 |
| **Inoculated** | 157 | 145.3 | 12.3 | 4.7 | 2.8 | 23.3 | 15.6 | 204.2 |
| **Control** | 157 | 63.5 | 7.5 | 1.0 | 0.2 | 13.3 | 3.61 | 89.2 |
| **Inoculated** | 158 | 159.9 | 5.5 | 4.3 | 2.6 | 10.1 | 10.2 | 192.6 |
| **Control** | 158 | 161.9 | 11.5 | 4.3 | 2.3 | 12.1 | 9.5 | 201.7 |
| **Inoculated** | 170 | 161.0 | 17.5 | 4.5 | 2.5 | 25.2 | 12 | 222.8 |
| **Control** | 170 | 154.1 | 9.7 | 3.9 | 1.8 | 15.5 | 8.6 | 193.8 |
| **Inoculated** | 176 | 236.3 | 23.4 | 7.3 | 5.0 | 35 | 19.9 | 327.0 |
| **Control** | 176 | 163.4 | 25.9 | 4.5 | 3.4 | 14.4 | 14.4 | 226.1 |
| **Inoculated** | 178 | 316.5 | 30.8 | 9.7 | 6.0 | 31 | 24.6 | 418.6 |
| **Control** | 178 | 146.1 | 35.5 | 8.9 | 4.7 | 30.7 | 20.4 | 246.3 |
| **Inoculated** | 180 | 189.2 | 10.0 | 5.3 | 3.2 | 21.1 | 12.8 | 241.8 |
| **Control** | 180 | 69.6 | 7.2 | 1.7 | 0.9 | 8.3 | 5.5 | 93.5 |
| **Inoculated** | 183 | 177.4 | 21.3 | 5.8 | 4.5 | 26.6 | 17.5 | 252.9 |
| **Control** | 183 | 122.6 | 12.0 | 3.5 | 2.6 | 16 | 10.7 | 167.5 |
| **Inoculated** | 188 | 100.8 | 10.6 | 3.0 | 2.6 | 17.9 | 9.4 | 144.5 |
| **Control** | 188 | 97.39 | 19.3 | 3.2 | 2.91 | 22.9 | 12.8 | 158.5 |
| **Inoculated** | 190 | 150.3 | 21.5 | 4.8 | 4.5 | 25.3 | 15.8 | 222.2 |
| **Control** | 190 | 131.2 | 17.6 | 3.9 | 3.53 | 12.4 | 11.4 | 180.1 |
| **Inoculated** | 195 | 145.8 | 23.0 | 4.7 | 4.51 | 24 | 17 | 219.1 |
| **Control** | 195 | 132.4 | 17.4 | 3.7 | 3.1 | 18.1 | 13 | 187.8 |
| **Inoculated** | 201 | 185.1 | 27.3 | 5.2 | 4.6 | 19.9 | 17.1 | 259.2 |
| **Control** | 201 | 141.9 | 30.9 | 4.9 | 6.7 | 21.4 | 22 | 228 |
| **Inoculated** | 203 | 134.7 | 14.9 | 4.2 | 4.1 | 18.5 | 16.6 | 193.1 |
| **Control** | 203 | 81.5 | 6.9 | 2.7 | 4.4 | 9.7 | 14.6 | 120 |
| **Inoculated** | 1586 | 204.1 | 24.4 | 7.3 | 10.2 | 14.4 | 30.5 | 290.9 |
| **Control** | 1586 | 95.0 | 21.7 | 3.902 | 6.08 | 2.75 | 19.7 | 149.1 |
| **Inoculated** | 1917 | 108.3 | 24.7 | 3.5 | 4.4 | 11.6 | 14.2 | 166.71 |
| **Control** | 1917 | 98.8 | 16.0 | 2.6 | 3.9 | 8.3 | 13.7 | 143.5 |
| **Inoculated** | 1918 | 176.8 | 22.5 | 6.5 | 7.0 | 15.6 | 21.9 | 250.5 |
| **Control** | 1918 | 93.7 | 12.7 | 2.5 | 3.7 | 7.7 | 12.3 | 132.8 |
| **Inoculated** | 1924 | 72.3 | 16.8 | 1.7 | 1.8 | 6.9 | 7.1 | 106.6 |
| **Control** | 1924 | 50.2 | 7.7 | 0.6 | 1.5 | 0.6 | 5.6 | 66.4 |
| **Inoculated** | 1951 | 76.4 | 20.1 | 2.4 | 2.6 | 11.1 | 9.5 | 122.2 |
| **Control** | 1951 | 67.5 | 7.3 | 1.5 | 1.6 | 8.1 | 7.0 | 93.2 |
| **Inoculated** | 1952 | 168.8 | 24.2 | 6.1 | 7.9 | 24.1 | 31.4 | 262.8 |
| **Control** | 1952 | 105.1 | 28.5 | 3.5 | 4.6 | 8.3 | 15.5 | 165.6 |
| **Inoculated** | 1976 | 178.3 | 20.4 | 5.5 | 6.2 | 15.2 | 20.6 | 246.3 |
| **Control** | 1976 | 141.0 | 13.1 | 4.3 | 3.9 | 17.2 | 16 | 195.6 |
| **Inoculated** | 1978 | 115.3 | 20.3 | 3.7 | 3.5 | 18.3 | 14.7 | 175.9 |
| **Control** | 1978 | 131.8 | 16.1 | 4.4 | 5.3 | 14.2 | 18.7 | 190.5 |
| **Inoculated** | 1980 | 73.2 | 7.6 | 1.7 | 2.5 | 12.2 | 10.9 | 108.3 |
| **Control** | 1980 | 112.0 | 30.2 | 3.4 | 3.3 | 10.1 | 12.7 | 171.7 |
| **Inoculated** | 1986 | 156.4 | 26.7 | 5.4 | 5.1 | 21.2 | 19.8 | 234.8 |
| **Control** | 1986 | 98.0 | 8.5 | 3.1 | 3.39 | 7.96 | 12.2 | 133.3 |
| **Inoculated** | 2105 | 203.8 | 29.1 | 6.7 | 2.05 | 25.1 | 14.6 | 281.4 |
| **Control** | 2105 | 147.0 | 21.6 | 4.6 | 0.8 | 13.7 | 7.2 | 195.0 |
| **Inoculated** | 2106 | 183.2 | 16.8 | 5.8 | 1.1 | 21.8 | 6.9 | 235.8 |
| **Control** | 2106 | 111.7 | 13.6 | 3.3 | 0.1 | 12.1 | 3.0 | 144.0 |
| **Inoculated** | 2113 | 176.9 | 27.2 | 5.8 | 1.9 | 14.2 | 9.0 | 235.2 |
| **Control** | 2113 | 152.1 | 32 | 4.8 | 1.0 | 7.3 | 5.2 | 202.6 |
